# Conjugative Transfer of a Novel Staphylococcal Plasmid Encoding the Biocide Resistance Gene, *qacA*

**DOI:** 10.3389/fmicb.2018.02664

**Published:** 2018-11-19

**Authors:** Patrick T. LaBreck, Gregory K. Rice, Adrian C. Paskey, Emad M. Elassal, Regina Z. Cer, Natasha N. Law, Carey D. Schlett, Jason W. Bennett, Eugene V. Millar, Michael W. Ellis, Theron Hamilton, Kimberly A. Bishop-Lilly, D. Scott Merrell

**Affiliations:** ^1^Department of Microbiology and Immunology, Uniformed Services University of the Health Sciences, Bethesda, MD, United States; ^2^Naval Medical Research Center, Biological Defense Research Directorate, Fort Detrick, MD, United States; ^3^Leidos, Reston, VA, United States; ^4^Henry M. Jackson Foundation for the Advancement of Military Medicine, Rockville, MD, United States; ^5^Infectious Diseases Clinical Research Program, Department of Preventive Medicine and Biostatistics, Uniformed Services University of the Health Sciences, Bethesda, MD, United States; ^6^Martin Army Community Hospital, Fort Benning, GA, United States; ^7^Walter Reed Army Institute of Research, Silver Spring, MD, United States; ^8^Department of Medicine, Uniformed Services University of the Health Sciences, Bethesda, MD, United States; ^9^University of Toledo College of Medicine and Life Sciences, Toledo, OH, United States

**Keywords:** antiseptic, Chlorhexidine digluconate, *Staphylococcus aureus*, plasmid acquisition, conjugation

## Abstract

*Staphylococcus aureus* is the leading cause of skin and soft tissue infections (SSTI). Some *S. aureus* strains harbor plasmids that carry genes that affect resistance to biocides. Among these genes, *qacA* encodes the QacA Multidrug Efflux Pump that imparts decreased susceptibility to chlorhexidine, a biocide used ubiquitously in healthcare facilities. Furthermore, chlorhexidine has been considered as a *S. aureus* decolonization strategy in community settings. We previously conducted a chlorhexidine-based SSTI prevention trial among Ft. Benning Army trainees. Analysis of a clinical isolate (C02) from that trial identified a novel *qacA*-positive plasmid, pC02. Prior characterization of *qacA*-containing plasmids is limited and conjugative transfer of those plasmids has not been demonstrated. Given the implications of increased biocide resistance, herein we characterized pC02. *In silico* analysis identified genes typically associated with conjugative plasmids. Moreover, pC02 was efficiently transferred to numerous *S. aureus* strains and to *Staphylococcus epidermidis*. We screened additional *qacA*-positive *S. aureus* clinical isolates and pC02 was present in 27% of those strains; other unique *qacA*-harboring plasmids were also identified. Ten strains were subjected to whole genome sequencing. Sequence analysis combined with plasmid screening studies suggest that *qacA*-containing strains are transmitted among military personnel at Ft. Benning and that strains carrying *qacA* are associated with SSTIs within this population. The identification of a novel mechanism of *qacA* conjugative transfer among Staphylococcal strains suggests a possible future increase in the prevalence of antiseptic tolerant bacterial strains, and an increase in the rate of infections in settings where these agents are commonly used.

## Importance

QacA is known to decrease bacteria susceptibility to chlorhexidine. Given the profound implications of increased resistance to chlorhexidine, it is vital to understand the mechanisms that contribute to the spread of *qacA* among bacteria. Analysis of a clinical *S. aureus* isolate revealed the first direct evidence of horizontal transfer of *qacA* across strains; transfer was mediated via a conjugative plasmid, pC02. In addition to transfer of *qacA* to multiple *S. aureus* strains, the plasmid was also conjugated to other members of the Staphylococcus genus. The *S. aureus* strain that harbors the *qacA*-positive conjugative plasmid was associated with a SSTI cluster among military trainees. The identification of *qacA* on a conjugative plasmid within a clinically successful strain stresses the importance of continued surveillance for *qacA* in *S. aureus* clinical isolates and underscores current efforts for increased antiseptic stewardship.

## Introduction

Community-Associated Methicillin-resistant *Staphylococcus aureus* (CA-MRSA) infections are a significant issue in the United States, and it is estimated that they are responsible for 65% of all MRSA infections (Dukic et al., 2013). In contrast to Healthcare-Associated MRSA (HA-MRSA), which typically only infects individuals with comorbidities, CA-MRSA is a common cause of skin and soft tissue infections (SSTI) in healthy populations. While overall CA-MRSA rates are climbing, certain populations are disproportionately affected, including groups that live in confined settings, such as inmates and military trainees (Aiello et al., 2006; Beauparlant, 2016; Boivin et al., 2016).

Asymptomatic colonization with *S. aureus* is common; ~30% of healthy individuals harbor *S. aureus* in the nares and as many as 20% of these individuals are persistently colonized (Kluytmans et al., 1997; Wertheim et al., 2005). Colonization of multiple body sites is known to occur (Albrecht et al., 2015; Singh et al., 2016), and colonization is a clear risk factor for *S. aureus* infection (Albrecht et al., 2015). Given these possibilities, decolonization of *S. aureus* has been employed as a disease prevention strategy (Buehlmann et al., 2008; Coates et al., 2009). Indeed, antimicrobials such as mupirocin and/or chlorhexidine can be administered to high-risk patients as a means to decolonize these individual and to decrease the potential for subsequent infections; several studies have shown decolonization with these agents as a successful means of reducing HA-MRSA infections (Buehlmann et al., 2008; Septimus and Schweizer, 2016). These successful colonization interventions within the hospital setting have prompted calls for a similar approach for the prevention of CA-MRSA (Fritz et al., 2011; Ellis et al., 2014; Karanika et al., 2016; Tidwell et al., 2016).

Two interventional trials among military trainees previously evaluated the effectiveness of chlorhexidine against SSTI (Morrison et al., 2013; Ellis et al., 2014). The trial among Army trainees at Fort Benning, Georgia specifically examined the effectiveness of a once-weekly use of chlorhexidine-based body wash (HibiClens) (Ellis et al., 2014). Trainees in groups randomized to use chlorhexidine received a bottle of 4% chlorhexidine soap to use for their extra weekly shower. Participants were monitored for SSTI, and those with purulent SSTI had abscess and nasal swabs taken. The swabs were used for bacterial culture and isolates of *S. aureus* were collected.

Given the concern that exposure to any antimicrobial can select for resistant isolates, each of the isolates was subsequently screened for increased chlorhexidine resistance as defined by the positive detection of *qacA/B* by real-time PCR; these genes encode for the QacA/B efflux pumps that are associated with decreased susceptibility to chlorhexidine. Of 615 isolates, 1.6% were shown to be *qacA/B*-positive (Schlett et al., 2014). This prevalence of *qacA/B* positive isolates is comparable to previous prevalence reports within North America (Longtin et al., 2011; Fritz et al., 2012; Popovich et al., 2014). However, the prevalence of *qacA/B* strains varies greatly and rates in North America are considerably lower than in Europe and Asia (Mayer et al., 2001; Noguchi et al., 2005; Ghasemzadeh-Moghaddam et al., 2014). The high rates of *qacA/B* presence in those areas of the world lead to concerns about the implication of bacteria with decreased susceptibility to biocides like chlorhexidine. Furthermore, while true “resistance” to chlorhexidine has yet to be observed, it is possible that the presence of *qacA* may provide a fitness advantage *in-vivo* to sub-optimal concentrations of chlorhexidine (Madden and Sifri, 2018).

Various studies have sought to understand the ability of individual Qac efflux pumps to mediate decreased susceptibility to antiseptics. For example, the QacA efflux pump has been shown to confer protection against quaternary ammonium compounds and to divalent organic cations like chlorhexidine. Conversely, while QacB is highly similar to QacA and is also part of the same major facilitator superfamily (MFS), QacB appears to offer little/no protection to divalent organic cations (Paulsen et al., 1996). The other Qac efflux pumps (QacC-QacJ and QacZ) are part of the Small Multidrug Transporter (SMR) family and each have various effects on antiseptic resistance (Furi et al., 2013; Wassenaar et al., 2015). The genes encoding the Qac efflux pumps are located on plasmids, which impacts possible mechanisms of spread of these genes across strains. For example, *qacC*, which is also known as *smr*, was previously found to be carried on conjugative plasmids as well as on small rolling circle plasmids (Littlejohn et al., 1991; Morton et al., 1993; Berg et al., 1998). Furthermore, transduction has been shown to facilitate transfer of plasmid-born *qacB* across strains. Conversely, *qacA* has only been found on large non-conjugative multidrug resistance plasmids; these plasmids lack the transfer, or *tra* genes, that are required for conjugative transfer (Tennent et al., 1989; McCarthy and Lindsay, 2012). As a result, horizontal transfer of *qacA* has not previously been documented (Nakaminami et al., 2007). Thus, it is not clear how or whether *qacA* is able to be horizontally spread across *S. aureus* strains, and if so, whether such spread could contribute to the prevalence of this factor in the *S. aureus* population.

Among the *qacA/B* positive strains obtained from the SSTI prevention trial (Ellis et al., 2014), we previously characterized a single clinical isolate, 2014.C02 (C02), obtained from a case of SSTI in the chlorhexidine study group (Johnson et al., 2015). C02 showed a significant decrease in susceptibility to chlorhexidine and whole genome sequencing (WGS) revealed a novel 61.5 kb plasmid (pC02) that harbored *qacA*. Intriguingly, this plasmid showed limited homology to the prototypical *qacA*-positive plasmid (pSK1) that is found among *S. aureus* strains (Jensen et al., 2010). Sequence analysis of pC02 identified genes for resistance to cadmium, antiseptics, β-lactams and erythromycin (Johnson et al., 2015). Furthermore, the annotation of pC02 revealed potential conjugation genes. Given that horizontal transfer of *qacA* has not previously been demonstrated, we explored the ability of *qacA* to be mobilized across *S. aureus* strains. Herein we report the first evidence of conjugative transfer of *qacA* and show that this transfer can also occur to other members of the Staphylococcus genus; conjugal transfer was sufficient to mediate decreased susceptibility to chlorhexidine in the recipient strains. Detailed characterization of additional *qacA*/B clinical isolates that were identified in the study revealed several strains that harbored pC02, presenting the intriguing possibility that natural horizontal transfer of pC02 and concomitant reduced susceptibility to chlorhexidine may facilitate strain survival in this population.

## Materials and methods

### Bacterial strains, plasmids, and growth conditions

All the bacterial strains and plasmids used in this study are listed in Table 1. Strains were cultured in BBL™ Muller Hinton II (Cation-Adjusted) Broth (MHB) or Trypic Soy Broth (TSB) (Becton Dickinson, Franklin Lakes, NJ). For solid media, broth was supplemented with 1.7% agar (Alfa Aesar, Waltham, MA). Where needed, strains were cured of their plasmid as described in Udo et al. (1997). Briefly, a culture was grown overnight shaking at 37°C and then diluted 1:500 in TSB. This culture was grown at 42°C for 48 h and dilutions were then plated on TSA and grown at 37°C to obtain single colonies. Plates containing isolated colonies were replica plated to MHA and MHA supplemented with 20 μg/mL of cadmium chloride and then grown at 37°C. Cadmium sensitive colonies were double purified and screened with the pC02 PCR screen described below to test for the presence of the large plasmid(s). Large plasmid cured strains were made resistant to rifampin and novobiocin as previously described by selecting mutant strains that were resistant to rifampin (Sigma-Aldrich, St Louis, MO) followed by subsequent passage in increasing concentrations of novobiocin (Sigma) (Udo et al., 1997).

**Table 1:** Bacterial strains used in the study.

**Strain name^a^**	**Lab strain designation**	**Description^b^**	**Battalion and Company**	**Platoon^c^**	**Collection Date**	**Study Group^d^**	**Accession number of whole genome**	**MLST^e^**	**Antibiotic Susceptibility^f^**	**Real-time PCR Results^g^**	**References**
*S.aureus* 2014.C01 (C01)	DSM1417	CI	A 2-58	4	12-Jul-11	CHG	CP012119.2	ST8	Ery^R^,Lvx^R^,Oxa^R^,Sxt^s^, Cli^s^	*femA, mecA, pvl*	(Johnson et al., 2015)
*S. aureus* 2014.C02 (C02)	DSM1418	CI	A 2-58	4	21-Sep-11	CHG	CP012120.2	ST8	Ery^R^,Lvx^R^,Oxa^R^,Sxt^S^, Cli^S^	*qacA, femA, mecA, pvl*	(Johnson et al., 2015)
*S. aureus* 1626.N	DSM1487	N	A 2-54	2	11-Mar-11	S	Not Sequenced	ST8	Ery^R^,Lvx^S^,Oxa^R^,Sxt^S^, Cli^S^	*qacA, femA, mecA, mupA, pvl*	This study
*S. aureus* 2148.N	DSM1488	N	B 1-19	4	13-Oct-11	ES	CP016856.2	ST72	Ery^S^,Lvx^S^,Oxa^S^,Sxt^S^, Cli^S^	*femA, pvl*	This study
*S. aureus* 5107.N	DSM1489	N	F 3-330	4	26-Jul-10	S	Not Sequenced	?	Ery^R^,Lvx^S^,Oxa^R^,Sxt^S^, Cli^S^	*femA, mecA, pvl*	This study
*S. aureus* 5116.N	DSM1490	N	A 1-50	1	26-Jul-10	ES	Not Sequenced	ST8	Ery^R^,Lvx^S^,Oxa^R^,Sxt^S^, Cli^S^	*femA, mecA*	This study
*S. aureus* 5118.N	DSM1491	N	E 3-330	2	26-Jul-10	S	CP016855.2	ST8	Ery^R^,Lvx^S^,Oxa^R^,Sxt^S^, Cli^S^	*qacA, femA, mecA*	This study
*S. aureus* 3011.C01	DSM1493	CI	B 1-50	-	17-Mar-11	ES	Not Sequenced	ST8	Ery^R^,Lvx^R^,Oxa^R^,Sxt^S^, Cli^S^	*femA, mecA, pvl*	this study
*S. aureus* 3020.C01	DSM1494	CI	A 2-58	-	19-Jul-11	CHG	CP025495.1	ST8	Ery^R^,Lvx^R^,Oxa^R^,Sxt^S^, Cli^S^	*qacA, femA, mecA, pvl*	This study
*S. aureus* 1971.C01	DSM1495	CI	C 2-54	3	8-Sep-11	S	CP016858.2	ST8	Ery^R^,Lvx^R^,Oxa^R^,Sxt^S^, Cli^S^	*qacA, femA, mecA, mupA, pvl*	This study
*S. aureus* 1624.C01	DSM1496	CI	A 2-54	2	11-Mar-11	S	Not Sequenced	ST8	Ery^R^,Lvx^S^,Oxa^R^,Sxt^S^, Cli^S^	*qacA, femA, mecA, mupA, pvl*	This study
*S. aureus* 1626.C01	DSM1497	CI	A 2-54	2	11-Mar-11	S	MCID00000000.2	ST8	Ery^R^,Lvx^S^,Oxa^R^,Sxt^S^, Cli^S^	*qacA, femA, mecA, mupA, pvl*	This study
*S. aureus* 1625.C01	DSM1498	CI	E 2-19	3	11-Mar-11	CHG	CP016863.2	ST8	Ery^R^,Lvx^S^,Oxa^R^,Sxt^S^, Cli^S^	*femA, mecA, pvl*	this study
*S. aureus* 2148.C01	DSM1499	CI	B 1-19	4	11-Oct-11	ES	CP017094.2	ST8	Ery^R^,Lvx^S^,Oxa^S^,Sxt^S^, Cli^S^	*qacA, femA, pvl*	This study
*S. aureus* 1534.C01	DSM1500	CI	B 2-58	1	29-Oct-10	CHG	Not Sequenced	ST8	Ery^R^,Lvx^R^,Oxa^R^,Sxt^S^, Cli^S^	*femA, mecA, pvl*	This study
*S. aureus* 2075.N02	DSM1501	N	B 2-58	4	19-Sep-11	CHG	Not Sequenced	ST8	Ery^R^,Lvx^S^,Oxa^S^,Sxt^S^, Cli^S^	*qacA, femA, pvl*	This study
*S. aureus* 2116.N02	DSM1502	N	B 2-58	4	28-Sep-11	CHG	Not Sequenced	ST8	Ery^R^,Lvx^S^,Oxa^S^,Sxt^S^, Cli^S^	*qacA, femA, pvl*	This study
*S. aureus* 1960.N02	DSM1503	N	C 2-19	1	1-Sep-11	CHG	Not Sequenced	ST72	Ery^S^,Lvx^S^,Oxa^S^,Sxt^S^, Cli^R^	*femA*	This study
*S. aureus* 1969.N	DSM1504	N	A 2-58	4	7-Sep-11	CHG	CP016861.2	ST8	Ery^R^,Lvx^R^,Oxa^R^,Sxt^S^, Cli^S^	*qacA, femA, mecA, pvl*	This study
*S. aureus* C02-RN	DSM1553	Large plasmid-cured strain *S.aureus* C02, Cadmium sensitive, Rifampin and Novobiocin resistant							Ery^S^, Cad^S^, Rif^R^, Nov^R^	NT	This study
*S. aureus* 42-RN	DSM1573	*S. aureus* RN4220, Cadmium sensitive, Rifampin and Novobiocin resistant							Ery^S^, Cad^S^, Rif^R^, Nov^R^	NT	This study
*S. aureus* 25-RN	DSM1575	Large plasmid-cured 1625.C01, Cadmium sensitive, Rifampin and Novobiocin resistant							Ery^S^, Cad^S^, Rif^R^, Nov^R^	NT	This study
*S. capitis* RN	DSM1581	Erythromycin sensitive, Rifampin and Novobiocin resistant							Ery^S^, Cad^R^, Rif^R^, Nov^R^	NT	This study
*S. epidermidis* RP62A-RN	DSM1582	Cadmium sensitive, Rifampin and Novobiocin resistant							Ery^S^, Cad^R^, Rif^R^, Nov^R^	NT	(Gill et al., 2005)
*E. faecalis* OG1-RF	DSM1580	Erythromycin sensitive, Rifampin and Fusidic acid resistant							Ery^S^, Cad^R^, Rif^R^, Fus^R^	NT	(Dunny et al., 1978)
*S. aureus* C02-RN TC	DSM1671	Transconjugant obtained from 2014.C02 to C02-RN mating							Cad^R^, Rif^R^, Nov^R^	NT	This study
*S. aureus* C02-RN TC2	DSM1672	Transconjugant obtained from 1969.N to C02-RN mating							Cad^R^, Rif^R^, Nov^R^	NT	This study
*S. aureus* 42-RN TC	DSM1673	Transconjugant obtained from 2014.C02 to 42-RN mating							Cad^R^, Rif^R^, Nov^R^	NT	This study
*S. capitis* RN TC	DSM1674	Transconjugant obtained from 2014.C02 to *S. capitis* RN mating							Ery^R^, Rif^R^, Nov^R^	NT	This study

a *RN indicates rifampin novobiocin resistant lab generated strain. RF indicates rifampin and fusidic acid resistant lab generated strain*.

b *CI, Clinical Isolate; N, Nasal colonizing Isolate*.

c *^−^, no data available*.

d *CHG, Chlorhexidine. S, Standard. ES, Enhanced Standard. Standard indicates a preventative medical briefing and access to an SSTI clinic. Enhanced standard indicates the standard intervention, an additional shower each week and enhanced SSTI education and surveillance. Chlorhexidine indicates receipt of a bottle of 4% chlorhexidine soap to use for the extra weekly shower in addition to the interventions received in the enhanced standard group*.

e *MLST, Multilocus Sequence Type; ?, Novel sequence type*.

f *^R^, resistant; ^S^, susceptible; Ery, erythromycin; Lvx, levofloxacin; Oxa, Oxacillin; Sxt, Sulfamethoxazole/Trimethoprim; Cli, Clindamycin; Cad, Cadmium; Rif, Rifampicin; Novo, Novobiocin; Fus, Fusidic acid. All clinical isolates were susceptible to linezolid, doxycycline, rifampin, vancomycin, gentamicin, and ceftaroline*.

g *genes shown to be present in strains by real-time PCR. NT, not tested*.

### PFGE, real-time PCR, antibiotic susceptibility testing

The strains isolated from Ft. Benning were globally characterized to define pulse field type, gene presence and antibiotic resistance patterns as previously described (Schlett et al., 2014).

### Sequencing

DNA extraction for Pacbio sequencing was performed from overnight liquid cultures using phenol-chloroform extraction with the Easy-DNA kit (Invitrogen, Waltham, MA). The DNA samples were sent to the University of Maryland Institute for Genome Sciences and sequenced with Pacbio RSII (Pacific Biosciences, Menlo Park, CA).

DNA extraction for Illumina sequencing was performed from overnight liquid cultures using the Wizard Kit (Promega, Madison, WI) and libraries were produced using the Nextera XT DNA Library Preparation Kit (Illumina, Inc., San Diego, CA) according to the manufacturer's instructions. Libraries were multiplexed and sequenced using an Illumina MiSeq 600-cycle kit and 2 × 300 base pair read lengths. Sequence read quality was analyzed with FastQC (Andrews, 2016) and low-quality bases were trimmed with Sickle (Joshi and Fass, 2011). Illumina and Pacbio sequence reads were assembled using SPAdes (Bankevich et al., 2012) and plasmidSPAdes (Antipov et al., 2016). The resulting contig sequences were compared to each other, to published reference genomes, and to PCR, Sanger sequencing, and agarose gel electrophoresis results of restriction enzyme digested and non-digested DNA in order to correctly assign contigs as chromosomal or plasmid as well as to look for assembly artifacts.

In order to determine the closest published reference for single nucleotide variation (SNV) analysis, the longest contig from each assembly was aligned against the National Center for Biotechnology Information (NCBI) nucleotide database using BLAST (Altschul et al., 1990). Based on these results, *S. aureus* USA300 strain TCH1516 (NCBI accession number CP000730) was selected as the reference. SNV data were analyzed using methods previously described (Millar et al., 2017). Briefly, the Bacterial and Archaeal Genome Analyzer (Williams, 2016) a wrapper for proven third-party bioinformatics tools, was used. Sequence reads were mapped to the reference using BWA (Li and Durbin, 2009), and variant calls and filtering were performed with GATK (McKenna et al., 2010). Genomic regions that contained insertions/deletions (indels), potential chromosomal rearrangements, and sequence repeats known to increase the likelihood of false-positive variant calls were excluded from the SNV set. A multiple sequence alignment (MSA) was created from nucleotide substitutions, small deletions called by GATK, and putative large deletions detected in the BWA sequence alignments where no reads mapped. A maximum likelihood tree was constructed from the nucleic acid MSA using PhyML (version 3.2) (Guindon et al., 2010) tree search with the GTR substitution model. Areas in the MSA containing structural variants (indels) were masked in the PHYLIP representation of the MSA as undetermined nucleotides and excluded from consideration in phylogenetic tree construction. A full list of indels and SNVs are available as Supplemental Tables 1, 2, respectively.

Gene content comparisons among strains were conducted using RAST for gene annotation and visualization (Aziz et al., 2008; Overbeek et al., 2014; Brettin et al., 2015).

### Chlorhexidine susceptibility testing

The minimum inhibitory concentration (MIC) of chlorhexidine for each strain was tested as previously described (Johnson et al., 2015). Briefly, ~1.5 × 10^5^ colony forming units (CFU) were inoculated into MHB containing increasing concentrations of purified (≥99.5%) chlorhexidine (Sigma). The cultures were grown at 37°C degrees overnight, shaking at 220 rpm. The MIC was determined by visual analysis of the culture's turbidity. Graphs were generated with Prism Software (GraphPad Software Inc., La Jolla, CA) with three independent replicates.

### DNA extraction, pC02 plasmid screening, MLST sequencing

*S. aureus* strains were grown overnight in MHB and 1.5 mL of each sample was centrifuged to pellet the cells. Cell pellets were treated with 0.1 mg of lysostaphin for 30–45 min at 37°C and plasmid DNA was purified using the QIAprep Spin Miniprep Kit (Qiagen, Germantown, MD). Chromosomal DNA was purified using the Wizard Genomic DNA Purification Kit (Promega) after lysostaphin treatment of cells for 30–45 min.

The primer sequences used to PCR amplify the backbone of pC02 are listed in Supplemental Table 3. Initially, primers were designed to be evenly spaced around the pC02 plasmid but the original primer set that annealed in the 40,000 kb region yielded multiple products. Thus, these primers were redesigned, shifted upstream and became primer-pair 7. The PCRs were performed in 25 μL reactions containing nuclease-free water, plasmid DNA, GoTaq Green Master Mix (Promega), and 0.3 μM each of the forward and reverse primers (Integrated DNA Technologies, Coralville, IA). PCR cycling conditions were as follows: incubation at 95°C for 180 s, 28 cycles of 95°C for 30 s, 53°C for 30 s and 72°C for 160 s followed by a final extension of 72°C for 5 min. The reaction amplicons were separated and visualized on 1% agarose gels.

**Figure 1:**
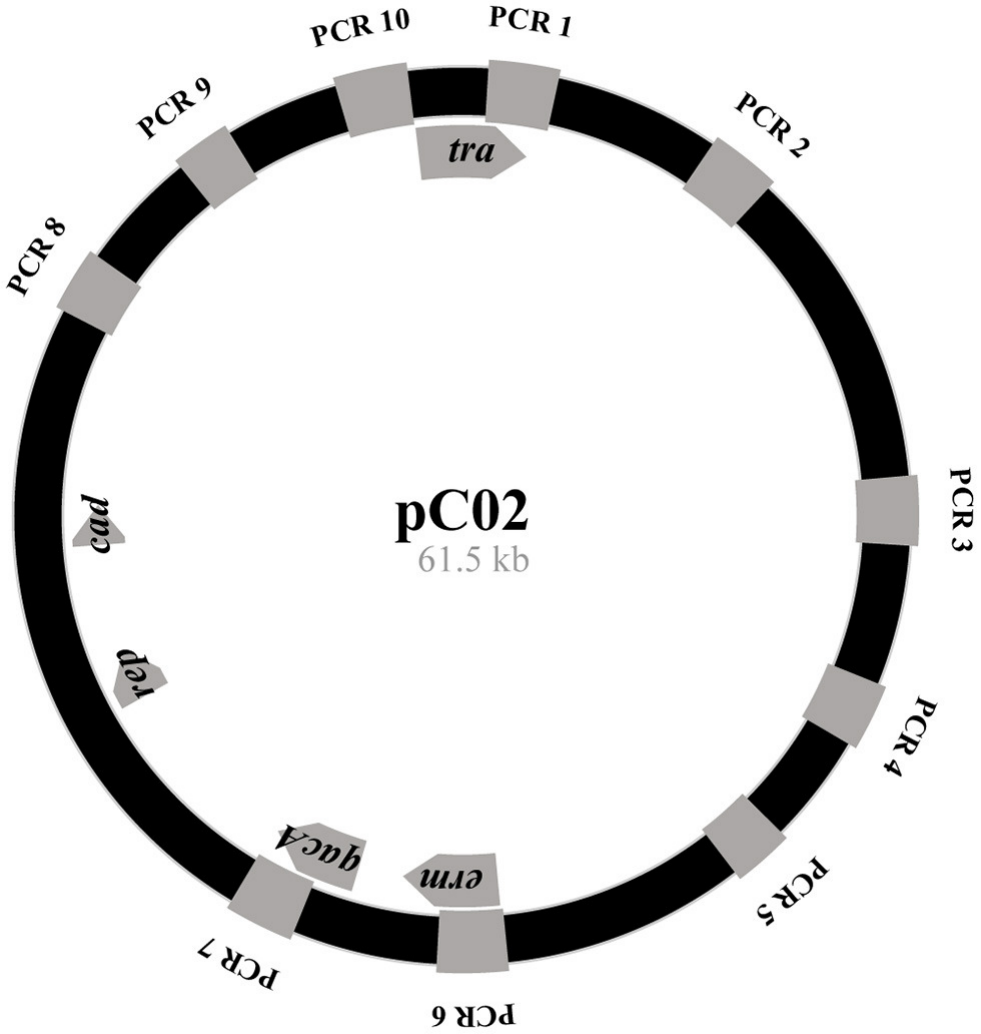
Simplified pC02 plasmid map. Regions amplified during the pC02 PCR screen are indicated by gray boxes along the plasmid backbone. Genes of interest are labeled in the gray arrows: *cad* (cadmium resistance loci), *tra* (putative conjugation gene) *erm* (erythromycin resistance loci), *qacA* (*qacA/R* loci) *rep* (replication A). Image drawn to scale.

For strains that were not subjected to WGS, the *qacA/B* gene was amplified and a portion was sequenced as previously described (Johnson et al., 2015). Utilized primers are listed in Supplemental Table 3. The plasmid map image (Figure 1) was generated with AngularPlasmid http://angularplasmid.vixis.com (AngularPlasmid, 2015).

MLST was performed as previously described (Enright et al., 2000). The trimmed sequences of *arc, aroE, glpF, gmk, pta, tpi, yqi*, were submitted to the MLST website: http://saureus.mlst.net/ (Imperial College in London, United Kingdom) for typing.

### Conjugative transfer

Filter-mating experiments were completed similar to a previous description (Forbes and Schaberg, 1983). Briefly, for *S. aureus*, recipient and donor cells were grown overnight in MHB and then subcultured 1:50 in MHB the next day. The cells were grown to an OD_600_ of 0.3–0.4 and 0.25 mL of both the recipient and donor cultures were then added to 2.5 mL of prewarmed MHB. The cells were then collected on a 0.45 μm filter and placed cell-side down on prewarmed Muller Hinton II (Cation-Adjusted) Agar (MHA). The mating mixtures were incubated overnight at 37°C and the following morning, filters were removed from the agar and vortexed vigorously for 1 min in 1.5 mL of PBS to release the cells from the filter. 10 μL of the cell suspension was taken to determine the recipient CFU and the remaining cells were plated onto selective media. Recipient CFU were enumerated on MHA supplemented with 5 μg/mL of novobiocin sodium salt (Sigma) and 10 μg/mL of rifampicin (Sigma). The transconjugants were identified after 48–72 h of growth on MHA supplemented with 5 μg/mL novobiocin, 10 μg/mL of rifampicin and 20 μg/mL of cadmium chloride. For experiments where *Staphylococcus epidermidis* was the recipient, numbers of recipient cells were determined by plating on TSA supplemented with 5 μg/mL novobiocin sodium salt and 10 μg/mL rifampicin and transconjugants were enumerated after 48 h of growth on TSA supplemented with 5 μg/mL of novobiocin sodium salt, 10 μg/mL of rifampicin and 10 μg/mL of erythromycin. For conjugation experiments where *Enterococcus faecalis* was the recipient, MHB/MHA were substituted with TSB/Trypic Soy Agar (TSA), respectively, in order to obtain similar growth between *S. aureus* and *E. faecalis*. Numbers of recipient cells were determined by plating on TSA supplemented with 10 μg/mL fusidic acid sodium salt (Sigma) and 10 μg/mL rifampicin. The transconjugants were identified after 48 h of growth on TSA supplemented with 10 μg/mL of fusidic acid sodium salt, 10 μg/mL of rifampicin and 10 μg/mL of erythromycin (Sigma). In all cases, the conjugation frequency was determined by dividing the number of transconjugants by the number of recipient cells. The reported frequencies are the average of at least three independent experiments. To confirm that transconjugants actually contained the pC02 plasmid, a subset of the isolates were restreaked onto their appropriate selective media, and then screened by colony PCR using primer-pair 7 of the pC02 screen.

### Transduction control assays

The possibility of movement of pC02 by phage was tested in several possible ways. First, the filter-mating protocol was conducted with the following modifications: 0.22 μm filter-sterilized supernatants (0.250 mL) from the C02 donor were mixed with 0.250 mL of the C02-RN recipient cells in 2.5 mL of MHB. This mixture was incubated at 30°C for 90 min, and then cells were collected on a 0.45 μm filter. Following collection on the 0.45 μm filter, the assay proceeded as described for the other filter-mating experiments.

Secondly, the necessity of cell-to-cell contact for conjugation was tested using a modified filter-mating assay. Briefly, C02 and C02-RN were grown to an OD_600_ of 0.3 to 0.4. 0.25 mL of both the C02-RN recipient and C02 donor cultures were individually added to separate culture flasks containing 2.5 mL of prewarmed MHB. The donor and recipient cells were individually collected on separate 0.45 μm filters. The recipient cell filter was placed cell-side-down on MHA and the donor cell filter was placed on top of the recipient filter with the cells adjacent to the back of the recipient filter. At the end of the mating assay, both of the filters were removed and vortexed together in 1.5 mL of PBS and processed as described above. Finally, to examine the possibility of any endogenous phage release, donor cells were also pretreated with mitomycin C (Sigma) to induce any possible phage. Overnight cultures of C02 were diluted 1:100 in 10 mL of TSB and grown at 37°C to an OD_600_ of 0.6–0.8. 1.0–2.5 μg/mL of mitomycin C was then added to the cultures and induction was allowed to proceed at 30°C for 6–8 h before cultures were placed at 4°C for 16 h. Afterwards, the cultures were filter sterilized through a 0.45 μm filter and then serial diluted in phage buffer (Novick, 1963). Resident phage were titrated by serial dilution with RN4220 on TSA plates with TSB soft agar (0.5%). No plaques were ever obtained.

## Results

### Initial characterization of *qacA/B*-positive isolates

A previous molecular epidemiologic study used WGS to identify a clonal HA-MRSA outbreak strain that contained a *qacA*-positive plasmid that may have given the strain a fitness advantage within the hospital environment (Senn et al., 2016). However, there is a lack of genomic information available for CA-MRSA strains that carry *qacA*. To characterize both colonizing and clinically relevant *qacA*-positive *S. aureus* strains, we initially examined 11 *qacA*/B positive strains and 8 *qacA*/B-negative control strains (Tables 1, 2) that were all obtained from a chlorhexidine-based SSTI prevention trial (Ellis et al., 2014; Schlett et al., 2014); the previously characterized C01 (*qacA*-) and C02 (*qacA*+) strains (Johnson et al., 2015) were included for comparison (Table 1). These *S. aureus* strains were collected from all three study groups (chlorhexidine, standard and enhanced standard-see Table 1 footnote d) from July 2010 through October 2011. All of the strains were USA300 with the exception of one Methicillin-Sensitive *Staphylococcus aureus* (MSSA) strain (2148.N), which had a pulse field-type that was indistinguishable. A majority of the strains were from two MLST lineages (ST8, ST72) and one strain (5107.N) had a previously uncharacterized sequence type due to a novel *aroE* allele. Antibiotic susceptibility testing revealed erythromycin resistance was common in the strains, occurring in 16/18 (88%) strains. Additionally, one strain (1960.N02) was also resistant to clindamycin. All tested strains were susceptible to sulfamethoxazole. Moreover, *mupA*, which is required for high-level resistance to mupirocin, was identified in four strains that contained *qacA/B*.

**Table 2:** pC02-based PCR screen.

**Strains^a^**	**Study Arm^b^**	**1^c^**	**2**	**3**	**4**	**5**	**6**	**7**	**8**	**9**	**10**	***qacA***	**Average CHG MIC μg/mL^d^**	**Median CHG MIC μg/ml of plasmid-pattern group^e^**
**REFERENCE STRAINS**														
2014.C01^*^	CHG	–	–	–	–	–	+	–	+	–	–	–	**0.33**	
2014.C02^*^	CHG	+	+	+	+	+	+	+	+	+	+	+	**0.90**	
**STRAINS FROM SSTI PREVENTION TRIAL**														
1960.N02^$^	CHG	–	–	–	–	–	–	–	–	–	–	–	**0.20**	**0.22 (0.20–0.23)**
2148.N^$^^*^	ES	–	–	–	–	–	–	–	–	–	–	–	**0.23**	
1534.C01	CHG	–	–	–	–	–	+	–	+	–	–	–	**0.30**	
1625.C01	CHG	–	–	–	–	–	+	–	+	–	–	–	**0.30**	
3011.C01	ES	–	–	–	–	–	+	–	+	–	–	–	**0.20**	**0.30 (0.20–0.33)**
5107.N	S	–	–	–	–	–	+	–	+	–	–	–	**0.27**	
5116.N	ES	–	–	–	–	–	+	–	+	–	–	–	**0.33**	
1624.C01	S	–	–	–	–	–	+	–	+	–	–	+	**1.03**	
1626.C01^*^	S	–	–	–	–	–	+	–	+	–	–	+	**1.07**	**1.05 (0.63–1.07)**
1626.N^*^	S	–	–	–	–	–	+	–	+	–	–	+	**1.07**	
1971.C01	S	–	–	–	–	–	+	–	+	–	–	+	**0.63**	
1969.N	CHG	+	+	+	+	+	+	+	+	+	+	+	**1.00**	**1.0 (0.90–1.03)**
3020.C01	CHG	+	+	+	+	+	+	+	+	+	+	+	**1.03**	
2075.N02^$^	CHG	–	–	–	–	–	+	+	+	–	–	+	**1.30**	
2116.N02^$^	CHG	–	–	–	–	–	+	+	+	–	–	+	**1.30**	**1.3 (1.10–1.37)**
2148.C01^$^^*^	ES	–	–	–	–	–	+	+	+	–	–	+	**1.37**	
5118.N	S	–	–	–	–	–	+	+	+	–	–	+	**1.10**	

a *$, MSSA isolate*.

* *multiple isolates were obtained from the same patient/numbers in the strain name indicate individual patient designations*.

b *CHG, chlorhexidine; ES, enhanced standard; S, standard*.

c *Number 1–10 indicate the primer sets used for the pC02 screen, qacA is an independent PCR for qacA/B. +, PCR amplification. - no PCR amplification*.

d *CHG, chlorhexidine; MIC, minimum inhibitory concentration*.

e *Group range and median*.

To specifically examine the *qacA/B*-positive strains for the presence of pC02, we developed a PCR based screen. A total of 10 primer-pairs were designed to span the entire pC02 sequence (Figure 1). Of these, the primers in primer-pair 7 annealed to *qacA/B* and to the pC02 backbone. Additionally, an internal *qacA/B* primer pair was also included in the screen. A total of 3/11 *qacA/B*-positive strains, including the C02 control, yielded positive PCR amplicons for all 10 primer-pairs within the pC02 screen, suggesting that these strains carry pC02 or a pC02-like plasmid (Table 2). In addition to the pC02-like amplicon pattern, among *qacA/B*-positive strains we also identified two additional amplicon patterns; 4/11 strains amplified with primer pairs 6, 8 and the internal *qacA/B* primers while 4/11 amplified with primer pairs 6, 7, 8 and the internal *qacA/B* primers. Among the *qacA*-negative strains, two major patterns were observed; five strains showed a pattern that was indistinguishable from the C01 isolate (amplicons with primer pairs 6 and 8, termed pC01-like) and two strains yielded no amplification products.

Given the functional data that indicate differences in the ability of QacA and QacB to mediate reduced susceptibility to chlorhexidine (Paulsen et al., 1996), we sequenced the *qacA/B* amplicon to distinguish *qacA* and *qacB*. Of the seven identified single nucleotide variants (SNVs) that discriminate *qacA* from *qacB*, a single SNV at residue 323 is responsible for an amino acid substitution that confers the substrate specificity for the efflux pumps (Paulsen et al., 1996). Sequencing analysis from the amplicon produced from the internal *qacA/B* primers (Supplemental Table 3) revealed that all of the strains encoded the *qacA* sequence at this key residue (data not shown). *En masse*, the data from this screen suggested that several different *qacA*-containing plasmid types were present in this study population and that pC02 or a pC02-like plasmid was present in clinical isolates that were obtained from multiple individuals.

### Chlorhexidine susceptibility testing

**Figure 2:**
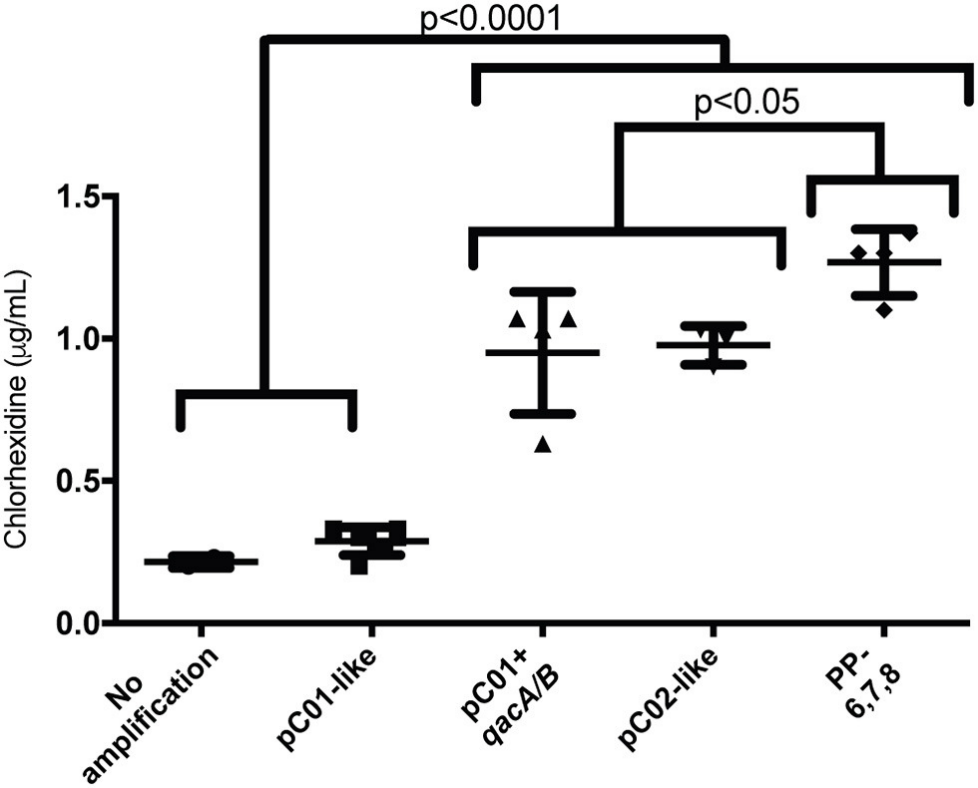
Chlorhexidine MIC for each plasmid-pattern group. The MIC of chlorhexidine for each strain was determined by broth dilution. All strains were grouped according to the results of the pC02 PCR screen as follows: No amplification, strains that didn't amplify at all with the pC02 primer set; pC01-like, strains that had an amplification pattern that matched the control 2014.C01 isolate (amplification with 6 and 8); pC01+*qacA/B*, strains that had an amplification pattern that matched 2014.C01 but also amplified with the *qacA/B* primer set; pC02-like, strains that had an amplification pattern that matched the control 2014.C02 strain; and PP-6,7,8, strains that showed amplification with primer sets 6, 7, 8, and *qacA/B*. Graphed values represent means from three independent replicates for each strain. Error bars represent the standard deviation of the group mean. Statistically significant differences are indicated as determined by a one-way Anova with correction for multiple comparisons using Tukey's multiple comparison method.

Given that the presence of *qacA* has been shown in some, but not all, cases to be associated with decreased susceptibility to chlorhexidine (Johnson et al., 2013; McDanel et al., 2013) and given that our sequencing results indicated that all of the *qac*-positive strains carried *qacA*, we next sought to determine whether these strains showed decreased susceptibility to chlorhexidine. Broth dilution assays using pure chlorhexidine revealed a 3–4 fold decrease in susceptibility to chlorhexidine in all strains that harbored *qacA* (Table 2). When the MIC values were plotted based on plasmid type (Table 2), all strains carrying *qacA* displayed a statistically significant decrease in susceptibility to chlorhexidine as compared to strains lacking *qacA* (Figure 2). Furthermore, the plasmid-pattern group (positive for primer-pair 6,7,8) displayed significantly higher resistance to chlorhexidine than all other strains that contained *qacA* (Figure 2). Taken together, these results indicate that *S. aureus* strains that carry *qacA* show a decreased susceptibility to chlorhexidine.

### Whole genome sequencing of selected clinical isolates

While MLST and PFGE profiles can provide fast preliminary information concerning strain relatedness, these techniques do not accurately determine strain clonality or provide detailed genomic information regarding the chromosome or mobile genetic elements, such as plasmids, which often carry resistance determinants and virulence genes (McCarthy and Lindsay, 2012). To further characterize the obtained *S. aureus* strains, eight strains were selected for WGS: two *qacA* negative strains, and two strains from every *qacA* positive plasmid-pattern group (Table 2). In addition, C01 and C02, which were both previously sequenced using PacBio technology (Johnson et al., 2015), were included for resequencing using MiSeq. To improve the accuracy of the assembled genomes, each strain was ultimately sequenced using both MiSeq and PacBio technologies. The average (min-max) read depth obtained with the MiSeq was 110 (range 67–167), whereas for the PacBio data the average (min-max) read depth was 17 (range 11–24). Analysis of the co-assembled Illumina short read and PacBio long read sequencing data revealed that each of the strains sequenced in this study had a similarly-sized chromosome, ranging in size from ~2.7 to 2.9 million base pairs. Additionally, each strain carried at least one, and in many cases, several plasmids of varying sizes. The various replicons and their sizes are presented in Table 3.

**Table 3:** Summary of *S. aureus* replicons.

**Strain (NCBI bioproject)**	**Chromosome Size (bp)**	**Plasmid Size (bp)**	**Plasmid Accession Number**	***qacA* (Y/N)**
*S. aureus* strain C01 (PRJNA287576)	2,917,985	pC01a 3,269	CP025490.1	N
		pC01b 27,043	CP012118.2	N
*S. aureus* strain C02 (PRJNA287791)	2,864,345	pC02a 3,269	CP025489.1	N
		pC02 61,539	CP012121.2	Y
*S. aureus* strain 1625.C01 (PRJNA321054)	2,877,915	p1625.C01 27,067	CP016862.2	N
*S. aureus* strain 1626.C01 (PRJNA321114)	2,871,639^*^	p1626.C01a 3,269	CM009343.1	N
		p1626.C01b 27,209	CM009344.1	N
		p1626.C01c 43,994	CM009345.1	Y
*S. aureus* strain 1969.N (PRJNA321120)	2,864,344	p1969.Na 3,269	CP025487.1	N
		p1969.Nb 61,505	CP016860.2	Y
*S. aureus* strain 1971.C01 (PRJNA321116)	2,873,208	p1971.C01a 3,269	CP025486.1	N
		p1971.C01b 21,841	CP016859.2	Y
		p1971.C01c 37,941	CP016857.2	N
*S. aureus* strain 2148.C01 (PRJNA321118)	2,856,605	p2148.C01a 3,266	CP025488.1	N
		p2148.C01b 32,660	CP017095.2	Y
*S. aureus* strain 2148.N (PRJNA321119)	2,748,803	p2148.N 3,459	CP025481.1	N
*S. aureus* strain 3020.C01 (PRJNA321117)	2,865,166	p3020.C01a 3,269	CP025496.1	N
		p3020.C01b 61,505	CP025497.1	Y
*S. aureus* strain 5118.N (PRJNA321122)	2,873,063	p5118.Na 3,269	CP025482.1	N
		p5118.Nb 53,799	CP016854.2	Y

* *Scaffolded assembly in multiple contigs*.

Overall, the 10 chromosomes sequenced in this study were found to be very similar to one another and to the reference strain USA300_TCH1516 (Highlander et al., 2007). However, a few interesting differences were observed. For example, gross inspection of chromosomal sequence alignments indicated three main areas of significant sequence variation with respect to the reference: a region in and around the gene encoding the replication initiation protein; a nearby region encoding a cassette chromosome recombinase A, a Zn-dependent hydroxyacylglutathione hydrolase/polysulfide binding protein, and some hypothetical proteins; and perhaps most significantly, a roughly 13 kilobase (kb) super-antigen encoding pathogenicity island (PAI; Supplemental Figure 1). This PAI, which is found in *S. aureus* USA300_TCH1516, was found to be absent in three of the 10 strains sequenced in this study. In strain USA300_TCH1516 and in seven of the strains described herein, this PAI encodes 17 genes, including genes for superantigen enterotoxin SEK, superantigen enterotoxin SEL, and secreted protein Ear. To verify that the apparent lack of this PAI in strains C02, 3020.C01, and 1969.N was not due to a missassembly, we performed BLAST against chromosomal and plasmid contigs as well as mapping of raw reads to the PAI sequence; in all cases the absence of the PAI was verified.

**Figure 3:**
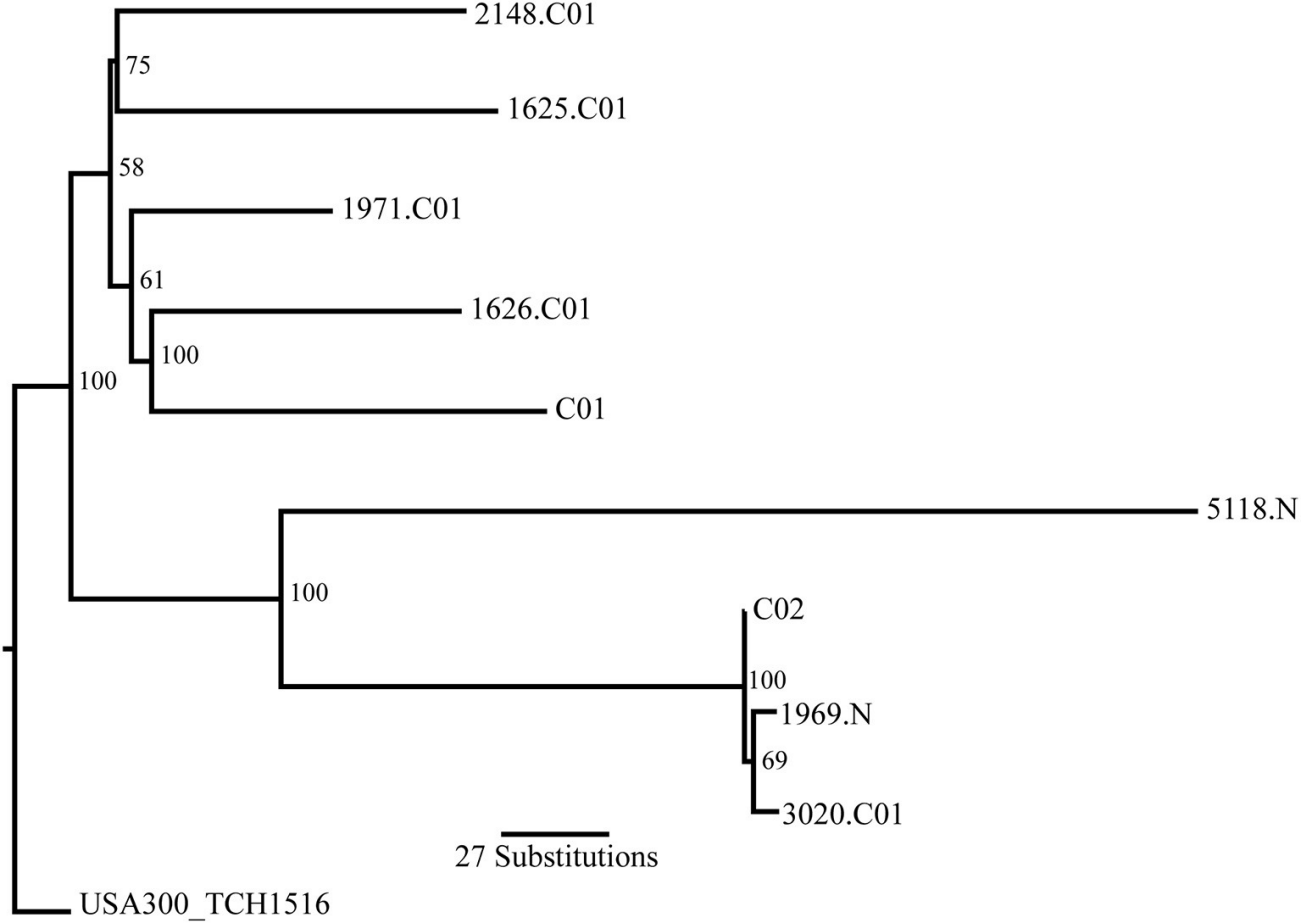
Dendrogram of MRSA isolates that were subjected to whole genome sequencing and analysis in this study. A multiple sequence alignment (MSA) was created from nucleotide substitutions. A maximum likelihood tree was constructed with *S. aureus* USA300_TCH1516 as the root from the nucleic acid MSA using PhyML (version 3.2) tree search with the GTR substitution model and 1000 bootstrap replicates. Bootstrap values are shown at the base of the individual nodes.

To examine relatedness of the strains, single nucleotide variation (SNV)-based phylogenies were constructed. Applying the GATK standard “hard” filter, and after filtering variants that occur in regions of genome repeats and putative chromosomal rearrangements, a total of 785 SNVs were identified (Supplemental Table 2). One of the nasal carriage isolates, 2148.N, was an outlier from the remainder of the strains and was subsequently removed from the phylogenetic tree that is presented in Figure 3; the original tree is presented in Supplemental Figure 2. Of note, the other two sequenced nasal carriage isolates clustered in a single clade with several SSTI isolates. Additionally of note, C02 and 3020.C01 were essentially clonal with the nasal carriage isolate 1969.N, perhaps reflecting the origin of these three isolates from the same battalion and company within the span of 2 months (Table 1). These three strains were also the only three sequenced strains in this study that were found to lack a PAI. Between C02 and 3020.C01, only six SNVs were identified, five of which were located in coding regions. Of those five, three were synonymous SNVs. Between 3020.C01 and 1969.N, eleven SNVs were identified, nine of which were in coding regions; of those nine, seven were synonymous. Between 1969.N and C02, seven SNVs were identified, six of which were located in coding regions; of those six, two were synonymous. Table 4 represents the SNVs by position (with respect to the coordinates of the reference genome *S. aureus* USA300_TCH1516; NC_010079.1) for each of the three isolates that were found to be clonal. The bulk of the SNVs were identified in a gene encoding an enoyl-ACP reductase. Of note, all of the SNVs in this gene were found to be synonymous substitutions. We hypothesize that that this may be due to the essential nature of the product of this gene, which is used to complete the fatty acid chain elongation cycle and is necessary for a properly functioning cell membrane (Chaudhuri et al., 2009). Specifically, it catalyzes the stereospecific reduction of a double bond between the C2 and C3 positions of the growing fatty acid chain (Priyadarshi et al., 2010).

**Table 4:** Identified SNVs in strains that harbored pC02.

**Position^a^**	**Gene product**	**C02**	**3020.C01**	**1969.N**
494641	Hypothetical protein			Non-synonymous SNV
704843	–		SNV (noncoding)	
1014474	Enoyl-ACP reductase	Synonymous SNV	Synonymous SNV	
1014480		Synonymous SNV	Synonymous SNV	
1014681		Ambiguous basecall	Synonymous SNV	
1014693		Ambiguous basecall	Synonymous SNV	
1014699			Synonymous SNV	
1014708			Synonymous SNV	
1466112	Thymidylate synthase		Non-synonymous SNV	Non-synonymous SNV
1466151			Non-synonymous SNV	Non-synonymous SNV
1567680	Minor structural protein			Non-synonymous SNV
2650114	Lantibiotic ABC transporter ATP-binding protein	Synonymous SNV		Synonymous SNV
2850131	-			SNV (noncoding)

a *Single nucleotide variations (SNVs) by position (coordinates with respect to the reference genome for methicillin-resistant MRSA USA300_TCH1516; NC_010079.1) for each of the three isolates that were found to be clonal*.

Consistent with the very similar chromosomal sequences for C02, 3020.C01, and 1969.N, these three strains also had similar plasmid profiles, each having one roughly 3 kb plasmid and one roughly 61 kb plasmid; all three strains were *qac*A-positive by PCR (Table 2). Comparison of the WGS data for all of the strains in this study, showed roughly three categories of plasmid (Table 3) (1) an ~3 kb plasmid, which was identified in nine of ten strains and found to have 65–100% nucleotide identity among the ten strains; (2) an ~27–32 kb plasmid, which was identified in four of ten strains and found to have 69–99% nucleotide identity; and (3) an ~61kb plasmid, which was identified in three of ten strains and found to have 99.78–99.94% nucleotide identity. Among the various plasmid size categories, the 3 kb plasmids were all cryptic plasmids with the exception of p2148.N, which harbored a predicted cadmium transporter. The 3kb plasmids all contained Rep_1 rolling circle replication initiation proteins (Kwong et al., 2017). Conversely, the large plasmids all carried theta replicating RepA_N domain replication initiation proteins. In addition to a RepA_N protein, p1626.C01c and p1971.C01c also harbored a Rep_2 and Rep_1 rolling circle replication initiation protein, respectively. The multiple replication proteins suggest a recent plasmid integration event with a small rolling circle replicating plasmid. The 27 kb plasmids were nearly identical and contained genes for macrolide, β-lactams and cadmium resistance. In addition to the resistance genes carried on the 27 kb plasmids, the 32 kb plasmid (p2148.C01b) harbored a 6.5 kb region containing *qacA* and an IS5/IS1182 family transposase. The 61kb pC02-like plasmids carried *qacA* and resistance genes for cadmium, macrolides and β-lactams. When the plasmids were grouped and analyzed based on *qacA* status and not size, we found that the plasmids that carried *qacA* ranged in size from 21 to 61 kb. As a whole, the large plasmids contained numerous insertion elements, including IS6, IS257, and IS5 transposases. Moreover, enzymes required for DNA mobilization, including invertases and resolvases, were found throughout the large plasmids. Indeed, examination of the plasmid population as a whole, suggested modularity in the staphylococcal plasmids where some segments were highly conserved between the various plasmid groups. Additionally, putative metabolic genes were identified in several plasmids, for example, p5118.Nb harbored two enzymes predicted to play a role in the ribulose monophosphate pathway for formaldehyde detoxification (Orita et al., 2006). *En masse*, these findings highlight the constant genetic flux that occurs between these extrachromosomal genetic molecules. Overall the genetic diversity seen across these plasmids likely contributes to the ability of Staphylococcus to adapt to a wide variety of environments and stresses.

### Conjugative transfer of the *qacA*-positive plasmid pC02

Previous sequence analysis of the *qacA*-containing pC02 plasmid identified a putative type IV secretion system (Johnson et al., 2015). To further investigate the pC02 components, the resequenced plasmid was re-annotated with the Rapid Annotation using Subsystem Technology (RAST) service (Aziz et al., 2008; Overbeek et al., 2014; Brettin et al., 2015). RAST revealed a putative TraG protein, which is commonly associated with conjugative plasmids (Firth et al., 1993; Morton et al., 1993). Although the exact function of TraG has yet to be identified in Gram-positive bacteria, it is believed to function as a coupling protein for the relaxase enzyme and the membrane bound secretion system (Cabezón et al., 1997). Additionally, RAST identified a putative conjugal ATPase, TraE, which is also known as VirB4. TraE helps supply the energy necessary for conjugative transfer (Walldén et al., 2012). The presence of two genes commonly associated with conjugation systems and the significance of possible conjugal transfer of *qacA*-containing plasmids in *S. aureus* led us to explore the conjugation potential of pC02. We questioned whether pC02 could be mobilized by conjugation from the parental C02 strain to a pC02 plasmid-cured derivative that was resistant to rifampin and novobiocin (C02-RN). Filter-mating assays with C02 (donor) and C02-RN (recipient) followed by selection of transconjugants on rifampin, novobiocin and cadmium (pC02 contains a cadmium resistance gene), revealed an average frequency of conjugation of 7.51 × 10^−9^ (Table 5). Moreover, the other *S. aureus* strains that harbored pC02 (3020.C01 and 1969.N) were able to transfer their plasmid to C02-RN at a similar frequency (Table 5). Transfer required cell-to-cell contact as transconjugants were never identified when the donor and recipient cells were separated by a filter.

**Table 5:** Conjugative transfer of pC02.

			**Conjugation Frequency**	
**Donor**	**Recipient**	**Conjugation**	**Average^a^**	**Standard Dev^b^**
C02	C02-RN	Yes	7.51E-09	6.58E-09
3020.C01	C02-RN	Yes	9.59E-09	5.39E-09
1969.N	C02-RN	Yes	1.42E-08	8.79E-09
C02	42-RN	Yes	8.20E-09	9.15E-09
3020.C01	42-RN	Yes	3.25E-09	3.98E-09
1969.N	42-RN	Yes	1.87E-09	5.83E-10
C02	1625-RN	Yes	1.29E-08	7.19E-09
3020.C01	1625-RN	Yes	3.95E-08	2.95E-08
1969.N	1625-RN	Yes	2.46E-09	1.91E-09
C02	*S. capitis* RN	Yes	6.82E-09	3.65E-09
C02	*S. epidermidis* RP62A-RN	Yes	7.25E-11	1.77E-10
C02	*E. faecalis* OG1-RF	Not observed	ND in 2.29E10	NA
1626.N	C02-RN	Not observed	ND in 1.26E10	NA
5118.N	C02-RN	Not observed	ND in 1.14E10	NA
1971.C01	C02-RN	Not observed	ND in 1.10E10	NA

a *ND, not detected in indicated number of examined recipient CFU*.

b *NA, not applicable*.

To examine the host range of pC02 conjugation, five additional recipient strains were tested: two *S. aureus* strains, two *S. epidermidis* strains and one *Enterococcus faecalis* strain. For the additional *S. aureus* strains, we chose the common lab strain RN4220 as well as a *qacA* minus strain from our collection (1625.C01). These two *S. aureus* strains both yielded transconjugates at a similar frequency to that seen with C02-RN. The *S. epidermidis* strains included a lab passaged strain (DSM1581) as well as strain RP62a, which is a recent clinical isolate (Gill et al., 2005). Again, transfer of pC02 to the lab strain occurred at a similar frequency to that seen with C02-RN. However, transfer to the clinical isolate was a very rare event; only a single transconjugant was ever detected for the *S. epidermidis* RP62a assays. Cross genera conjugation via mating with *E. faecalis* OG1-RF (Dunny et al., 1978) was not detected (Table 5). This conjugal transfer of pC02 was unique as the other *qacA*-positive plasmids found in strains 1626.N, 5118.N, and 1971.C01 were unable to be mobilized by conjugation; the lack of mobilization is likely no surprise given the absence of identifiable putative transfer genes on any of these other plasmids.

To ensure that pC02 was being mobilized by conjugation and not by phage-mediated transduction, supernatants collected from donor strains were mixed with recipient cells and the appearance of transductants was assessed; supernatants from overnight and log phase donor cultures were examined. No transductants were ever identified, suggesting that pC02 mobilization does occur via conjugation. Furthermore, to check for endogenous phage within C02, mitomycin C induction at various concentrations produced no visible plaques at a limit of detection of 100 plaque forming units.

**Figure 4:**
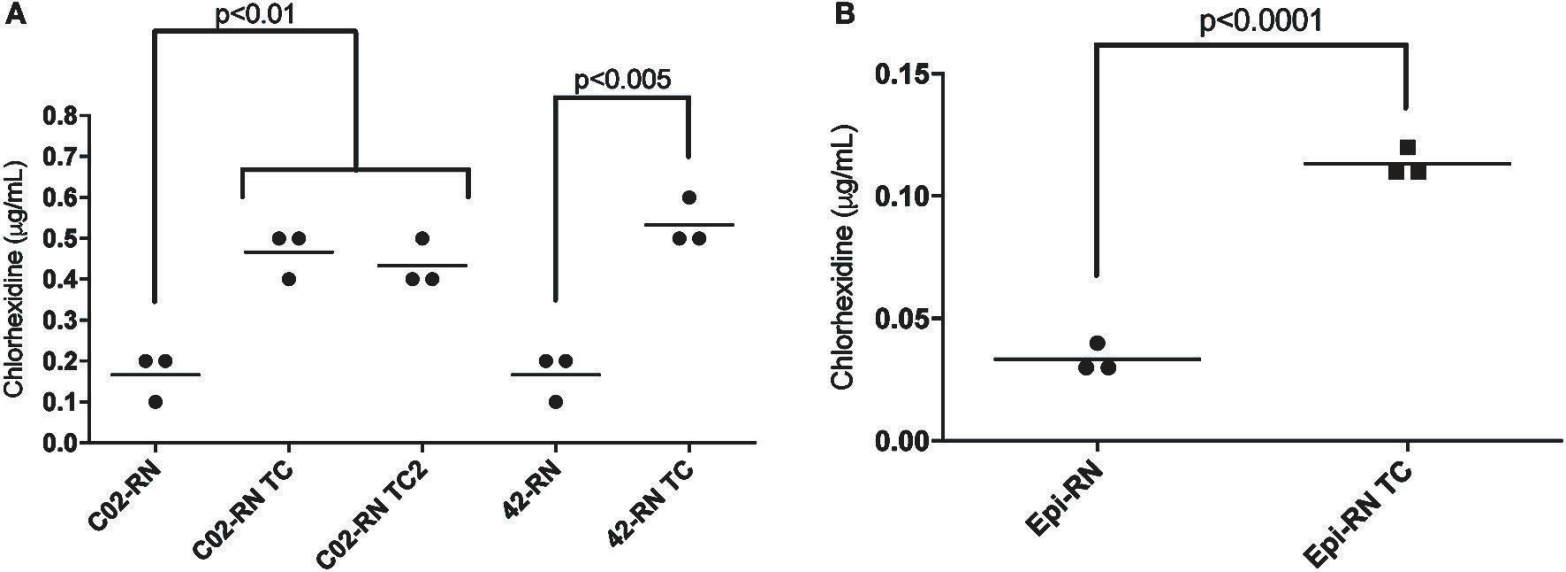
Chlorhexidine MIC for transconjugant strains. The MIC to chlorhexidine for each indicated strain was determined by broth dilution. **(A)** C02-RN TC is a transconjugant obtained from mating C02 with C02-RN. C02-RN TC2 is a transconjugant obtained from mating 1969.N with C02-RN. 42-RN TC is a transconjugant obtained from mating C02 with 42-RN. 42-RN and C02-RN are pC02-free recipient cells. **(B)** Epi-RN TC is a transconjugant obtained from mating C02 with *S. capitis* RN. Epi-RN is pC02-free recipient *S. capitis* RN. Graphed values represent three independent replicates for each strain and the bar indicates the mean. Statistically significant differences are indicated as determined by a Student's *t* test; a correction for multiple comparisons using the Holm-Sidak's method was applied for C02-RN, C02-RN TC and C02-RN TC2.

Given that *qacA* containing strains showed decreased susceptibility to chlorhexidine (Figure 2) and that pC02 was able to mobilize *qacA* to multiple recipient strains, we next asked whether conjugal transfer of pC02 was sufficient to affect chlorhexidine susceptibility of the recipient strains; we tested individual transconjugants from various matings for their susceptibility to chlorhexidine. Indeed, we found that all tested *S. aureus* pC02-containing transconjugants displayed an observable decrease in their susceptibility to chlorhexidine as compared to their respective parental strains (Figure 4A). This decrease in susceptibility was not only seen with the *S. aureus* transconjugants, but was also seen with *S. epidermidis*; the pC02-containing strain showed a 3-fold increase in chlorhexidine resistance as compared to the parental *S. epidermidis* strain (Figure 4B). Thus, transfer of pC02 and *qacA* was sufficient to impart decreased susceptibility to chlorhexidine in a susceptible strain background. *En masse*, our conjugation results show the first demonstration of conjugative transfer of *qacA* across *S. aureus* strains and to other Staphylococcal species; the downstream result of mobilization of *qacA* for the recipient cells was a decrease in susceptibility to chlorhexidine.

## Discussion

The dissemination of *qacA* among bacteria presents a potential risk to the efficacy of the highly employed chlorhexidine antiseptic. Mobile genetic elements are known for their ability to disseminate resistance elements quickly between bacteria. While *qacA* has been found in some *S. aureus* strains, it has previously only been found to exist on non-conjugative plasmids that are not easily horizontally disseminated to other strains. Our prior characterization of a clinical isolate that showed decreased susceptibility to chlorhexidine revealed a novel *qacA*-containing plasmid (pC02) that encoded possible conjugation genes (Johnson et al., 2015). Herein, we identified additional strains from Ft. Benning that carry pC02, showed that those strains represent an infectious clonal *qacA*-positive lineage and demonstrated the successful conjugal transfer of *qacA*-positive pC02 to multiple strains of *S. aureus* and to *S. epidermidis*.

The clonal nature of the pC02-containing strains C02, 1969.N and 3020.C01 was confirmed by WGS, which revealed very few SNVs among the three strains (Table 4). Of note, all three of these strains were isolated from different soldiers who were part of the same company (~200 soldiers). While two of the soldiers were part of the same platoon (a subgroup of a company comprised of ~50 soldiers), platoon data was not available for one individual (Table 1). Of these strains, 3020.C01 was isolated ~2 months before the other two strains. Moreover, while C02 and 3020.C01 were isolated from purulent abscesses, 1969.N was a nasal isolate. Thus, at a minimum pC02-containing strains can colonize or infect and can be spread among individuals in close contact. There are likely numerous opportunities for transmission of these strains, given that trainees train and live in the same physical environment for the 14-week training duration. It is worth noting that all three strains were isolated from individuals in groups randomized to receive chlorhexidine. Given that pC02 contains *qacA*, these strains may have been given a fitness advantage in the presence of routine (i.e., weekly) exposure to chlorhexidine. Similarly, an outbreak strain of ST228 MRSA was previously shown to harbor *qacA* in nearly all of the clinical isolates obtained from a tertiary care hospital in Switzerland (Senn et al., 2016); in the hospital environment, microbial exposure to chlorhexidine would likely be a constant, selective pressure. In a recent study, *S. epidermidis* isolates were collected from industrial cleanrooms and startlingly revealed to carry *qacA/B* at a high rate; 98.3% (56/57) of the isolates carried *qacA/B* (Ribič et al., 2017). This extreme example suggests that in environments where there is a constant exposure to chlorhexidine or other quaternary compounds, there is a strong selective pressure for Staphylococcal species to acquire and maintain *qacA/B*. While the specific advantage of CA-MRSA strains that contain *qacA* may be debatable, the increased usage of chlorhexidine in households and the increased incidence of CA-MRSA in hospital settings could provide the selection needed for rapid dispersal of *qacA* to various *Staphylococcal* species. Indeed, the demonstrated conjugal transfer of pC02 (Table 5) may represent one mechanism by which the prevalence of *qacA* may continue to increase.

It is worth noting that in addition to pC02 containing strains, we also identified and sequenced a variety of large (>21 kb) non-conjugative *qacA* positive plasmids. Furthermore, we showed that the presence of *qacA* on any of those plasmids was associated with a decreased susceptibility to chlorhexidine (Table 2 and Figure 2). Unexpectedly, one of the *qacA*-positive plasmid groups (PCR positive for region 6,7,8) showed a significantly higher MIC than the other *qacA*-positive plasmid groups. WGS sequence analysis of the two strains chosen for sequencing from that plasmid group did not reveal mutations in *qacA* or *qacR*, the latter of which encodes for the *qacA* transcriptional regulator, QacR. Thus, the increase in resistance to chlorhexidine displayed by these strains may be attributed to a higher copy number of these particular *qacA*-harboring plasmids or to additional differences that are particular to these strain backgrounds. In support of the importance of the strain background, we note that even though transconjugants that obtained pC02 showed decreased susceptibility to chlorhexidine (Figures 4A,B), the level of resistance was never as high as that seen in the original pC02 containing strains (Table 2 and Figure 2). Thus, additional strain specific components likely contribute to the overall level of chlorhexidine resistance imparted by the presence of *qacA*.

Compared to previously sequenced plasmids, the *qacA*-positive plasmids in our study showed overall sequence homology levels that ranged from 32 to 80%. Areas that showed homology to other plasmids typically had nearly 100% nucleotide identity while other areas showed virtually no conservation. This finding further supports the previously demonstrated modularity of Staphylococcal plasmids (McCarthy and Lindsay, 2012). Indeed, the high level of recombination and genetic exchange that is seen between Staphylococcal plasmids may assist in the selection of dominant clonal Staphylococcal strains that can then be responsible for outbreaks within hospitals and communities (DeLeo et al., 2010). The high levels of recombination seen between Staphylococcal plasmids in tandem with the constant exchange of genetic material via transduction, conjugation, and mobilization of non-conjugative plasmids highlights the overall genetic promiscuity of Staphylococcal plasmids (Deghorain and Van Melderen, 2012; McCarthy and Lindsay, 2012; O'Brien et al., 2015). While the role of the components carried on these modular plasmids is rather poorly characterized, presumably genes must impart some form of selection in various environmental niches. To this end, we note that within our population, *qacA* was often carried on plasmids that contained additional resistance determinants; this has also been observed in previous studies (Jensen et al., 2010; McCarthy and Lindsay, 2012). The importance of this finding lies in the fact that any selective pressure that selects for the presence of a single gene will also co-select for the stability of the other resistance genes found on large plasmids or other shared mobile elements (Carter et al., 2017). Thus, in hospitals and other environments where antiseptics are used liberally, residual chlorhexidine could drive an increase in multidrug resistant strains of Staphylococcus (Bjorland et al., 2005; Madden and Sifri, 2018). Importantly, the implication of chlorhexidine-driven co-selection is not only applicable to Staphylococcus; *qacA* has recently been identified in *Enterococcus faecalis, E. faecium, Acinetobacter baumannii*, and *Klebsiella pneumoniae* strains (Bischoff et al., 2012; Guo et al., 2015; Rizzotti et al., 2016; Liu et al., 2017).

While conjugative transfer of biocide resistance genes, such as *qacC*, has been previously shown (Lyon et al., 1987), prior to the work described herein, *qacA* had yet to be definitively identified on a conjugative element. Despite this, we do note one previous report that may indicate that *qacA* may have resided on a conjugative plasmid (pSAJ1) that was isolated in the 1980's from Japan (Yamamoto et al., 1988). The characterization of pSAJ1 that was conducted at that time showed restriction sites on the suspected antiseptic resistance gene that are very similar to the *qacA* sequence. Moreover, the authors showed that pSAJ1 is responsible for an antiseptic resistance profile that is similar to strains that carry *qacA*. While pSAJ1 was not sequenced and it is not clear if the plasmid did in fact carry *qacA*, the presence of *qacA* on a conjugative plasmid that was circulating in Asia in the 1980's could partially explain the very high prevalence of *qacA/B* found in current Staphylococcal strains from Asia (Wang et al., 2008a,b; Shamsudin et al., 2012; Cho et al., 2018). Currently, the rates of *qacA* positivity among community acquired *S. aureus* strains in the United States is fairly low: 0-1.6% (Fritz et al., 2012; Popovich et al., 2014; Schlett et al., 2014). However, given our herein described discovery of a *qacA*-containing plasmid that can be conjugated across *S. aureus* strains and even to some strains of *S. epidermidis*, one has to wonder if we are on the verge of observing a dramatic increase in the prevalence of *qacA* containing strains? Indeed, when one combines the ability of bacterial strains to move resistance components with the selective pressure imposed by indiscriminate use of antiseptics, it becomes almost too easy to envision a not so distant future where antiseptic resistance becomes a critical conversation. Clearly, continued surveillance and increased antiseptic stewardship are warranted for the continued clinical success of even antiseptics.

## Author contributions

PL, TH, KB-L, and DM conceived and designed the study. AP, and PL performed the sequencing and PL, GR, RC, and KB-L performed the bioinformatic analyses. CS, JB, EM, ME and NL designed and executed the original chlorhexidine intervention trial. PL, AP and EE processed and characterized the bacterial clinical isolates.

### Conflict of interest statement

The authors declare that the research was conducted in the absence of any commercial or financial relationships that could be construed as a potential conflict of interest.

## References

[B1] Aiello A. E. Lowy F. D. Wright L. N. Larson E. L. (2006). Meticillin-resistant *Staphylococcus aureus* among US prisoners and military personnel: review and recommendations for future studies. Lancet Infect. Dis. 6, 335–341. 10.1016/S1473-3099(06)70491-1 16728319

[B2] Albrecht V. S. Limbago B. M. Moran G. J. Krishnadasan A. Gorwitz R. J. McDougal L. K. (2015). *Staphylococcus aureus* colonization and strain type at various body sites among patients with a closed abscess and uninfected controls at US emergency departments. J. Clin. Microbiol. 53, 01371–01315. 10.1128/JCM.01371-15 PMC4609677 26292314

[B3] Altschul S. F. Gish W. Miller W. Myers E. W. Lipman D. J. (1990). Basic local alignment search tool. J. Mol. Biol. 215, 403–410. 10.1016/S0022-2836(05)80360-2 2231712

[B4] Andrews S. (2016). FastQC. Babraham Bioinformatics. Available online at: https://www.bioinformatics.babraham.ac.uk/projects/fastqc/ (Accessed November 2, 2016).

[B5] AngularPlasmid (2015). AngularPlasmid. Vixis. Available online at: http://angularplasmid.vixis.com/about.php (Accessed March 10, 2018).

[B6] Antipov D. Hartwick N. Shen M. Raiko M. Lapidus A. Pevzner P. (2016). plasmidSPAdes: assembling plasmids from whole genome sequencing data. Bioinformatics 32, 3380–3387. 10.1093/bioinformatics/btw493 27466620

[B7] Aziz R. K. Bartels D. Best A. A. DeJongh M. Disz T. Edwards R. A.. (2008). The RAST Server: rapid annotations using subsystems technology. BMC Genomics 9:75. 10.1186/1471-2164-9-75 18261238 PMC2265698

[B8] Bankevich A. Nurk S. Antipov D. Gurevich A. A. Dvorkin M. Kulikov A. S.. (2012). SPAdes: a new genome assembly algorithm and its applications to single-cell sequencing. J. Comput. Biol. 19, 455–477. 10.1089/cmb.2012.0021 22506599 PMC3342519

[B9] Beauparlant M. (2016). A metapopulation model for the spread of MRSA in correctional facilities. Infect. Dis. Model. 1, 11–27. 10.1016/j.idm.2016.06.001 29928718 PMC5963330

[B10] Berg T. Firth N. Apisiridej S. Hettiaratchi A. Leelaporn A. Skurray R. A. (1998). Complete nucleotide sequence of pSK41: evolution of staphylococcal conjugative multiresistance plasmids. J. Bacteriol. 180, 4350–4359. 9721269 10.1128/jb.180.17.4350-4359.1998 PMC107441

[B11] Bischoff M. Bauer J. Preikschat P. Schwaiger K. Mölle G. Hölzel C. (2012). First detection of the antiseptic resistance gene *qacA/B* in *Enterococcus faecalis*. Microb. Drug Resist. 18, 7–12. 10.1089/mdr.2011.0092 22017402

[B12] Bjorland J. Steinum T. Kvitle B. Waage S. Sunde M. Heir E. (2005). Widespread distribution of disinfectant resistance genes among staphylococci of bovine and caprine origin in Norway. J. Clin. Microbiol. 43, 4363–4368. 10.1128/JCM.43.9.4363-4368.2005 16145078 PMC1234083

[B13] Boivin M. Cowan D. Packnett E. Garvin N. (2016). AMSARA 2016 Annual Report. Available online at: http://www.amsara.amedd.army.mil/AMSARAAR.aspx (Accessed March, 2018).

[B14] Brettin T. Davis J. J. Disz T. Edwards R. A. Gerdes S. Olsen G. J.. (2015). RASTtk: a modular and extensible implementation of the RAST algorithm for building custom annotation pipelines and annotating batches of genomes. Sci. Rep. 5:8365. 10.1038/srep08365 25666585 PMC4322359

[B15] Buehlmann M. Frei R. Fenner L. Dangel M. Fluckiger U. Widmer A. (2008). Highly effective regimen for decolonization of methicillin-resistant *Staphylococcus aureus* carriers. Infect. Control Hosp. Epidemiol. 29, 510–516. 10.1086/588201 18510460

[B16] Cabezón E. Sastre J. I. De La Cruz F. (1997). Genetic evidence of a coupling role for the TraG protein family in bacterial conjugation. Mol. General Genet. 254, 400–406. 10.1007/s004380050432 9180693

[B17] Carter G. P. Schultz M. B. Baines S. L. da Silva A. G. Heffernan H. Tiong A. (2017). Topical antibiotic use co-selects for the carriage of mobile genetic elements conferring resistance to unrelated antimicrobials in *Staphylococcus aureus*. Antimicrob. Agents Chemother. 62:e02000-17. 10.1128/AAC.02000-17 PMC5786761 29229636

[B18] Chaudhuri R. R. Allen A. G. Owen P. J. Shalom G. Stone K. Harrison M. (2009). Comprehensive identification of essential *Staphylococcus aureus* genes using Transposon-Mediated Differential Hybridisation (TMDH). BMC Genomics 10:291 10.1186/1471-2164-10-291 19570206 PMC2721850

[B19] Cho O. H. Park K. H. Song J. Y. Hong J. M. Kim T. Hong S. I.. (2018). Prevalence and microbiological characteristics of *qacA/B*-positive methicillin-resistant *Staphylococcus aureus* isolates in a surgical intensive care unit. Microb. Drug Resist. 24, 283–289. 10.1089/mdr.2017.0072 28799881

[B20] Coates T. Bax R. Coates A. (2009). Nasal decolonization of *Staphylococcus aureus* with mupirocin: strengths, weaknesses and future prospects. J. Antimicrob. Chemother. 64, 9–15. 10.1093/jac/dkp159 19451132 PMC2692503

[B21] Deghorain M. Van Melderen L. (2012). The Staphylococci phages family: an overview. Viruses 4, 3316–3335. 10.3390/v4123316 23342361 PMC3528268

[B22] DeLeo F. R. Otto M. Kreiswirth B. N. Chambers H. F. (2010). Community-associated meticillin-resistant *Staphylococcus aureus*. Lancet 375, 1557–1568. 10.1016/S0140-6736(09)61999-1 20206987 PMC3511788

[B23] Dukic V. M. Lauderdale D. S. Wilder J. Daum R. S. David M. Z. (2013). Epidemics of community-associated methicillin-resistant *Staphylococcus aureus* in the United States: a meta-analysis. PLoS ONE 8:e52722. 10.1371/journal.pone.0052722 23300988 PMC3534721

[B24] Dunny G. M. Brown B. L. Clewell D. B. (1978). Induced cell aggregation and mating in *Streptococcus faecalis*: evidence for a bacterial sex pheromone. Proc. Natl. Acad. Sci. USA. 75, 3479–3483. 10.1073/pnas.75.7.3479 98769 PMC392801

[B25] Ellis M. W. Schlett C. D. Millar E. V. Wilkins K. J. Crawford K. B. Morrison-Rodriguez S. M.. (2014). Hygiene strategies to prevent methicillin-resistant *Staphylococcus aureus* skin and soft tissue infections: a cluster-randomized controlled trial among high-risk military trainees. Clin. Infect. Dis. 58, 1540–1548. 10.1093/cid/ciu166 24633684 PMC4017895

[B26] Enright M. C. Day N. P. Davies C. E. Peacock S. J. Spratt B. G. (2000). Multilocus sequence typing for characterization of methicillin-resistant and methicillin-susceptible clones of *Staphylococcus aureus*. J. Clin. Microbiol. 38, 1008–1015. 10698988 10.1128/jcm.38.3.1008-1015.2000 PMC86325

[B27] Firth N. Ridgway K. P. Byrne M. E. Fink P. D. Johnson L. Paulsen I. T.. (1993). Analysis of a transfer region from the staphylococcal conjugative plasmid pSK41. Gene 136, 13–25. 10.1016/0378-1119(93)90442-6 8293996

[B28] Forbes B. A. Schaberg D. R. (1983). Transfer of resistance plasmids from *Staphylococcus epidermidis* to *Staphylococcus aureus*: evidence for conjugative exchange of resistance. J. Bacteriol. 153, 627–634. 6822476 10.1128/jb.153.2.627-634.1983 PMC221678

[B29] Fritz S. A. Camins B. C. Eisenstein K. A. Fritz J. M. Epplin E. K. Burnham C.-A.. (2011). Effectiveness of measures to eradicate *Staphylococcus aureus* carriage in patients with community-associated skin and soft tissue infections: a randomized trial. Infect. Control Hosp. Epidemiol. 32, 872–880. 10.1086/661285 21828967 PMC3528015

[B30] Fritz S. A. Hogan P. G. Camins B. C. Ainsworth A. J. Patrick C. Martin M. S.. (2012). *Staphylococcus aureus* mupirocin and chlorhexidine resistance in patients with community-onset skin and soft tissue infections. Antimicrob. Agents Chemother. 57, 559–568. 10.1128/AAC.01633-12 23147738 PMC3535967

[B31] Furi L. Ciusa M. L. Knight D. Di Lorenzo V. Tocci N. Cirasola D.. (2013). Evaluation of reduced susceptibility to quaternary ammonium compounds and bisbiguanides in clinical isolates and laboratory-generated mutants of *Staphylococcus aureus*. Antimicrob. Agents Chemother. 57, 3488–3497. 10.1128/AAC.00498-13 23669380 PMC3719693

[B32] Ghasemzadeh-Moghaddam H. van Belkum A. Hamat R. A. van Wamel W. Neela V. (2014). Methicillin-susceptible and-resistant *Staphylococcus aureus* with high-level antiseptic and low-level mupirocin resistance in Malaysia. Microb. Drug Resist. 20, 472–477. 10.1089/mdr.2013.0222 24841796

[B33] Gill S. R. Fouts D. E. Archer G. L. Mongodin E. F. DeBoy R. T. Ravel J.. (2005). Insights on evolution of virulence and resistance from the complete genome analysis of an early methicillin-resistant *Staphylococcus aureus* strain and a biofilm-producing methicillin-resistant *Staphylococcus epidermidis* strain. J. Bacteriol. 187, 2426–2438. 10.1128/JB.187.7.2426-2438.2005 15774886 PMC1065214

[B34] Guindon S. Dufayard J. F. Lefort V. Anisimova M. Hordijk W. Gascuel O. (2010). New algorithms and methods to estimate maximum-likelihood phylogenies: assessing the performance of PhyML 3.0. Syst. Biol. 59, 307–321. 10.1093/sysbio/syq010 20525638

[B35] Guo W. Shan K. Xu B. Li J. (2015). Determining the resistance of carbapenem-resistant *Klebsiella pneumoniae* to common disinfectants and elucidating the underlying resistance mechanisms. Pathog. Glob. Health 109, 184–192. 10.1179/2047773215Y.0000000022 26184804 PMC4530556

[B36] Highlander S. K. Hultén K. G. Qin X. Jiang H. Yerrapragada S. Mason E. O.. (2007). Subtle genetic changes enhance virulence of methicillin resistant and sensitive *Staphylococcus aureus*. BMC Microbiol. 7:99. 10.1186/1471-2180-7-99 17986343 PMC2222628

[B37] Jensen S. O. Apisiridej S. Kwong S. M. Yang Y. H. Skurray R. A. Firth N. (2010). Analysis of the prototypical *Staphylococcus aureus* multiresistance plasmid pSK1. Plasmid 64, 135–142. 10.1016/j.plasmid.2010.06.001 20547176

[B38] Johnson J. G. Saye E. J. Jimenez-Truque N. Soper N. Thomsen I. Talbot T. R.. (2013). Frequency of disinfectant resistance genes in pediatric strains of methicillin-resistant *Staphylococcus aureus*. Infect. Control Hosp. Epidemiol. 34, 1326–1327. 10.1086/673983 24225622 PMC3965576

[B39] Johnson R. C. Schlett C. D. Crawford K. Lanier J. B. Merrell D. S. Ellis M. W. (2015). Recurrent methicillin-resistant *Staphylococcus aureus* cutaneous abscesses and selection of reduced chlorhexidine susceptibility during chlorhexidine use. J. Clin. Microbiol. 53, 3677–3682. 10.1128/JCM.01771-15 26292295 PMC4609685

[B40] Joshi N. A. Fass J. N. (2011). A Sliding-Window, Adaptive, Quality-Based Trimming Tool for FastQ files (Version 1.33) [Software]. Available online at: https://github.com/najoshi/sickle.

[B41] Karanika S. Kinamon T. Grigoras C. Mylonakis E. (2016). Colonization with methicillin-resistant *Staphylococcus aureus* and risk for infection among asymptomatic athletes: a systematic review and metaanalysis. Clin. Infect. Dis. 63, 195–204. 10.1093/cid/ciw240 27090988

[B42] Kluytmans J. van Belkum A. Verbrugh H. (1997). Nasal carriage of *Staphylococcus aureus*: epidemiology, underlying mechanisms, and associated risks. Clin. Microbiol. Rev. 10, 505–520. 10.1128/CMR.10.3.505 9227864 PMC172932

[B43] Kwong S. M. Ramsay J. P. Jensen S. O. Firth N. (2017). Replication of staphylococcal resistance plasmids. Front. Microbiol. 8:2279. 10.3389/fmicb.2017.02279 29218034 PMC5703833

[B44] Li H. Durbin R. (2009). Fast and accurate short read alignment with Burrows–Wheeler transform. Bioinformatics 25, 1754–1760. 10.1093/bioinformatics/btp324 19451168 PMC2705234

[B45] Littlejohn T. G. DiBerardino D. Messerotti L. J. Spiers S. J. Skurray R. A. (1991). Structure and evolution of a family of genes encoding antiseptic and disinfectant resistance in *Staphylococcus aureus*. Gene 101, 59–66. 10.1016/0378-1119(91)90224-Y 1840534

[B46] Liu W. J. Fu L. Huang M. Zhang J. P. Wu Y. Zhou Y. S.. (2017). Frequency of antiseptic resistance genes and reduced susceptibility to biocides in carbapenem-resistant *Acinetobacter baumannii*. J. Med. Microbiol. 66, 13–17. 10.1099/jmm.0.000403 27930267

[B47] Longtin J. Seah C. Siebert K. McGeer A. Simor A. Longtin Y.. (2011). Distribution of antiseptic resistance genes *qacA, qacB*, and *smr* in methicillin-resistant *Staphylococcus aureus* isolated in Toronto, Canada, from 2005 to 2009. Antimicrob. Agents Chemother. 55, 2999–3001. 10.1128/AAC.01707-10 21402848 PMC3101426

[B48] Lyon B. R. Gillespie M. T. Byrne M. E. May J. W. Skurray R. A. (1987). Plasmid-mediated resistance to gentamicin in *Staphylococcus aureus*: the involvement of a transposon. J. Med. Microbiol. 23:101. 10.1099/00222615-23-2-101 3031300

[B49] Madden G. R. Sifri C. D. (2018). Antimicrobial resistance to agents used for *Staphylococcus aureus* decolonization: is there a reason for concern? Curr. Infect. Dis. Rep. 20, 26. 10.1007/s11908-018-0630-0 29882094 PMC7015675

[B50] Mayer S. Boos M. Beyer A. Fluit A. C. Schmitz F.-J. (2001). Distribution of the antiseptic resistance genes *qacA, qacB* and *qacC* in 497 methicillin-resistant and-susceptible European isolates of *Staphylococcus aureus*. J. Antimicrob. Chemother. 47, 896–897. 10.1093/jac/47.6.896 11389128

[B51] McCarthy A. J. Lindsay J. A. (2012). The distribution of plasmids that carry virulence and resistance genes in *Staphylococcus aureus* is lineage associated. BMC Microbiol. 12:104 10.1186/1471-2180-12-104 22691167 PMC3406946

[B52] McDanel J. S. Murphy C. R. Diekema D. J. Quan V. Kim D. S. Peterson E. M.. (2013). Chlorhexidine and mupirocin susceptibilities of methicillin-resistant *Staphylococcus aureus* from colonized nursing home residents. Antimicrob. Agents Chemother. 57, 552–558. 10.1128/AAC.01623-12 23147721 PMC3535956

[B53] McKenna A. Hanna M. Banks E. Sivachenko A. Cibulskis K. Kernytsky A.. (2010). The genome analysis toolkit: a mapreduce framework for analyzing next-generation DNA sequencing data. Genome Res. 20, 1297–1303. 10.1101/gr.107524.110 20644199 PMC2928508

[B54] Millar E. V. Rice G. K. Elassal E. M. Schlett C. D. Bennett J. W. Redden C. L.. (2017). Genomic characterization of USA300 methicillin-resistant *Staphylococcus aureus* (MRSA) to evaluate intraclass transmission and recurrence of skin and soft tissue infection (SSTI) among high-risk military trainees. Clin. Infect. Dis. 65, 461–468. 10.1093/cid/cix327 28419202 PMC5849051

[B55] Morrison S. M. Blaesing C. R. Millar E. V. Chukwuma U. Schlett C. D. Wilkins K. J.. (2013). Evaluation of methicillin-resistant *Staphylococcus aureus* skin and soft-tissue infection prevention strategies at a military training center. Infect. Control Hosp. Epidemiol. 34, 841–843. 10.1086/671278 23838227 PMC5824622

[B56] Morton T. Eaton D. Johnston J. Archer G. (1993). DNA sequence and units of transcription of the conjugative transfer gene complex (trs) of *Staphylococcus aureus* plasmid pGO1. J. Bacteriol. 175, 4436–4447. 10.1128/jb.175.14.4436-4447.1993 7687249 PMC204884

[B57] Nakaminami H. Noguchi N. Nishijima S. Kurokawa I. So H. Sasatsu M. (2007). Transduction of the plasmid encoding antiseptic resistance gene *qacB* in *Staphylococcus aureus*. Biol. Pharmaceut. Bull. 30, 1412–1415. 10.1248/bpb.30.1412 17666795

[B58] Noguchi N. Suwa J. Narui K. Sasatsu M. Ito T. Hiramatsu K.. (2005). Susceptibilities to antiseptic agents and distribution of antiseptic-resistance genes *qacA/B* and *smr* of methicillin-resistant *Staphylococcus aureus* isolated in Asia during 1998 and 1999. J. Med. Microbiol. 54, 557–565. 10.1099/jmm.0.45902-0 15888465

[B59] Novick R. (1963). Analysis by transduction of mutations affecting penicillinase formation in *Staphylococcus aureus*. Microbiology 33, 121–136. 14072829 10.1099/00221287-33-1-121

[B60] O'Brien F. G. Yui Eto K. Murphy R. J. Fairhurst H. M. Coombs G. W. Grubb W. B.. (2015). Origin-of-transfer sequences facilitate mobilisation of non-conjugative antimicrobial-resistance plasmids in *Staphylococcus aureus*. Nucleic Acids Res. 43, 7971–7983. 10.1093/nar/gkv755 26243776 PMC4652767

[B61] Orita I. Sato T. Yurimoto H. Kato N. Atomi H. Imanaka T.. (2006). The ribulose monophosphate pathway substitutes for the missing pentose phosphate pathway in the archaeon *Thermococcus kodakaraensis*. J. Bacteriol. 188, 4698–4704. 10.1128/JB.00492-06 16788179 PMC1482999

[B62] Overbeek R. Olson R. Pusch G. D. Olsen G. J. Davis J. J. Disz T.. (2014). The SEED and the Rapid Annotation of microbial genomes using Subsystems Technology (RAST). Nucleic Acids Res. 42(Database issue), D206–D214. 10.1093/nar/gkt1226 24293654 PMC3965101

[B63] Paulsen I. T. Brown M. H. Littlejohn T. G. Mitchell B. A. Skurray R. A. (1996). Multidrug resistance proteins QacA and QacB from *Staphylococcus aureus*: membrane topology and identification of residues involved in substrate specificity. Proc. Natl. Acad. Sci. U.S.A. 93, 3630–3635. 10.1073/pnas.93.8.3630 8622987 PMC39662

[B64] Popovich K. J. Aroutcheva A. Hota B. Beavis K. G. Hayden M. K. Weinstein R. A. (2014). Anatomic sites of colonization with community-associated methicillin-resistant *Staphylococcus aureus*. Infect. Control Hosp. Epidemiol. 35, 1192–1194. 10.1086/677627 25111931 PMC4422190

[B65] Priyadarshi A. Kim E. E. Hwang K. Y. (2010). Structural insights into *Staphylococcus aureus* enoyl-ACP reductase (FabI), in complex with NADP and triclosan. Proteins Struct. Funct. Bioinform. 78, 480–486. 10.1002/prot.22581 19768684

[B66] Ribič U. Klančnik A. Jeršek B. (2017). Characterization of *Staphylococcus epidermidis* strains isolated from industrial cleanrooms under regular routine disinfection. J. Appl. Microbiol. 122, 1186–1196. 10.1111/jam.13424 28231617

[B67] Rizzotti L. Rossi F. Torriani S. (2016). Biocide and antibiotic resistance of *Enterococcus faecalis* and *Enterococcus faecium* isolated from the swine meat chain. Food Microbiol. 60, 160–164. 10.1016/j.fm.2016.07.009 27554158

[B68] Schlett C. D. Millar E. V. Crawford K. B. Cui T. Y. Lanier J. B. Tribble D. R.. (2014). Prevalence of chlorhexidine-resistant methicillin-resistant *Staphylococcus aureus* following prolonged exposure. Antimicrob. Agents Chemother. 58, 4404–4410. 10.1128/AAC.02419-14 24841265 PMC4136006

[B69] Senn L. Clerc O. Zanetti G. Basset P. Prod'hom G. Gordon N. C.. (2016). The stealthy superbug: the role of asymptomatic enteric carriage in maintaining a long-term hospital outbreak of ST228 methicillin-resistant *Staphylococcus aureus*. MBio 7, e02039–e02015. 10.1128/mBio.02039-15 26787833 PMC4725017

[B70] Septimus E. J. Schweizer M. L. (2016). Decolonization in prevention of health care-associated infections. Clin. Microbiol. Rev. 29, 201–222. 10.1128/CMR.00049-15 26817630 PMC4786886

[B71] Shamsudin M. Alreshidi M. Hamat R. Alshrari A. Atshan S. Neela V. (2012). High prevalence of *qacA/B* carriage among clinical isolates of meticillin-resistant *Staphylococcus aureus* in Malaysia. J. Hosp. Infect. 81, 206–208. 10.1016/j.jhin.2012.04.015 22633074

[B72] Singh J. Johnson R. C. Schlett C. D. Elassal E. M. Crawford K. B. Mor D.. (2016). Multi-body-site microbiome and culture profiling of military trainees suffering from skin and soft tissue infections at Fort Benning, Georgia. mSphere 1, e00232–e00216. 10.1128/mSphere.00232-16 27747300 PMC5064451

[B73] Tennent J. M. Lyon B. R. Midgley M. Jones G. Purewal A. S. Skurray R. A. (1989). Physical and biochemical characterization of the *qacA* gene encoding antiseptic and disinfectant resistance in *Staphylococcus aureus*. J. Gen. Microbiol. 135, 1–10. 2778425 10.1099/00221287-135-1-1

[B74] Tidwell J. Kirk L. Luttrell T. Pike C. A. (2016). CA-MRSA decolonization strategies: do they reduce recurrence rate? J. Wound Ostomy Continence Nurs. 43, 577–582. 10.1097/WON.0000000000000277 27820584

[B75] Udo E. E. Jacob L. Mokadas E. (1997). Conjugative transfer of high-level mupirocin resistance from *Staphylococcus haemolyticus* to other staphylococci. Antimicrob. Agents Chemother. 41, 693–695. 10.1128/AAC.41.3.693 9056015 PMC163773

[B76] Walldén K. Williams R. Yan J. Lian P. W. Wang L. Thalassinos K.. (2012). Structure of the VirB4 ATPase, alone and bound to the core complex of a type IV secretion system. Proc. Natl. Acad. Sci. U.S.A. 109, 11348–11353. 10.1073/pnas.1201428109 22745169 PMC3396474

[B77] Wang C. Cai P. Zhan Q. Mi Z. Huang Z. Chen G. (2008a). Distribution of antiseptic-resistance genes *qacA/B* in clinical isolates of meticillin-resistant *Staphylococcus aureus* in China. J. Hosp. Infect. 69, 393–394. 10.1016/j.jhin.2008.05.009 18602196

[B78] Wang J.-T. Sheng W.-H. Wang J.-L. Chen D. Chen M.-L. Chen Y.-C.. (2008b). Longitudinal analysis of chlorhexidine susceptibilities of nosocomial methicillin-resistant *Staphylococcus aureus* isolates at a teaching hospital in Taiwan. J. Antimicrob. Chemother. 62, 514–517. 10.1093/jac/dkn208 18477706

[B79] Wassenaar T. M. Ussery D. Nielsen L. N. Ingmer H. (2015). Review and phylogenetic analysis of *qac* genes that reduce susceptibility to quaternary ammonium compounds in Staphylococcus species. Eur. J. Microbiol. Immunol. 5, 44–61. 10.1556/EuJMI-D-14-00038 25883793 PMC4397847

[B80] Wertheim H. F. Melles D. C. Vos M. C. van Leeuwen W. van Belkum A. Verbrugh H. A.. (2005). The role of nasal carriage in *Staphylococcus aureus* infections. Lancet Infect. Dis. 5, 751–762. 10.1016/S1473-3099(05)70295-4 16310147

[B81] Williams D. (2016). Bacterial and Archaeal Genome Analyser [Online]. GitHub. Available online at: https://github.com/daveuu/baga. (Accessed November 2, 2016).

[B82] Yamamoto T. Tamura Y. Yokota T. (1988). Antiseptic and antibiotic resistance plasmid in *Staphylococcus aureus* that possesses ability to confer chlorhexidine and acrinol resistance. Antimicrob. Agents Chemother. 32, 932–935. 10.1128/AAC.32.6.932 3415214 PMC172311

