# Corrigendum: Conjugative Transfer of a Novel Staphylococcal Plasmid Encoding the Biocide Resistance Gene, *qacA*

**DOI:** 10.3389/fmicb.2020.00877

**Published:** 2020-05-14

**Authors:** Patrick T. LaBreck, Gregory K. Rice, Adrian C. Paskey, Emad M. Elassal, Regina Z. Cer, Natasha N. Law, Carey D. Schlett, Jason W. Bennett, Eugene V. Millar, Michael W. Ellis, Theron Hamilton, Kimberly A. Bishop-Lilly, D. Scott Merrell

**Affiliations:** ^1^Department of Microbiology and Immunology, Uniformed Services University of the Health Sciences, Bethesda, MD, United States; ^2^Naval Medical Research Center, Biological Defense Research Directorate, Fort Detrick, MD, United States; ^3^Leidos, Reston, VA, United States; ^4^Henry M. Jackson Foundation for the Advancement of Military Medicine, Rockville, MD, United States; ^5^Infectious Diseases Clinical Research Program, Department of Preventive Medicine and Biostatistics, Uniformed Services University of the Health Sciences, Bethesda, MD, United States; ^6^Martin Army Community Hospital, Fort Benning, GA, United States; ^7^Walter Reed Army Institute of Research, Silver Spring, MD, United States; ^8^Department of Medicine, Uniformed Services University of the Health Sciences, Bethesda, MD, United States; ^9^University of Toledo College of Medicine and Life Sciences, Toledo, OH, United States

**Keywords:** antiseptic, Chlorhexidine digluconate, *Staphylococcus aureus*, plasmid acquisition, conjugation

The authors wish to correct an improper bacterial species designation in the original article. Post publication, whole genome sequencing and targeted *rpoB* sequencing revealed that “*S. epidermidis* RN and S*. epidermidis* RN TC” are actually *Staphylococcus capitis*. Thus, all utilizations of “*S. epidermidis* RN and S*. epidermidis* RN TC” in the article should be replaced with “*S. capitis* RN” and “*S. capitis* RN TC”, respectively.

Additionally, a correction has been made to Keywords, (specific changes are underlined):

*Staphycoccus aureus* was changed to *Staphylococcus aureus* due to a misspelling.

A correction has been made to Keywords, (specific changes are underlined):

Chlorhexedine digluconate was changed to Chlorhexidine digluconate due to a misspelling.

A correction has been made to Introduction, Paragraph Number 5 (specific changes are underlined). In the original edited stage, a sentence was incorrectly pasted into the introduction. This sentence should be removed and has a strikethrough to designate the deletion.

Various studies have sought to understand the ability of individual Qac efflux pumps to mediate decreased susceptibility to antiseptics. For example, the QacA efflux pump has been shown to confer protection against quaternary ammonium compounds and to divalent organic cations like chlorhexidine. Conversely, while QacB is highly similar to QacA and is also part of the same major facilitator superfamily (MFS), QacB appears to offer little/no protection to divalent organic cations (Paulsen et al., 1996). The other Qac efflux pumps (QacC-QacJ and QacZ) are part of the Small Multidrug Transporter (SMR) family and each have various effects on antiseptic resistance (Furi et al., 2013; Wassenaar et al., 2015). The genes encoding the Qac efflux pumps are located on plasmids, which impacts possible mechanisms of spread of these genes across strains. For example, *qacC*, which is also known as *smr*, was previously found to be carried on conjugative plasmids as well as on small rolling circle plasmids (Littlejohn et al., 1991; Morton et al., 1993; Berg et al., 1998). Furthermore, transduction has been shown to facilitate transfer of plasmid-born *qacB* across strains. The corrected sentence should read. Conversely, *qacA* has only been found on large non-conjugative multidrug resistance plasmids; these plasmids lack the transfer, or *tra* genes, that are required for conjugative transfer (Tennent et al., 1989; McCarthy and Lindsay, 2012). As a result, horizontal transfer of *qacA* has not previously been documented (Nakaminami et al., 2007). Thus, it is not clear how or whether *qacA* is able to be horizontally spread across *S. aureus* strains, and if so, whether such spread could contribute to the prevalence of this factor in the *S. aureus* population.

The authors apologize for this error and state that this does not change the scientific conclusions of the article in any way. The original article has been updated.
