# Supplementary material for: Conjugative Transfer of a Novel Staphylococcal Plasmid Encoding the Biocide Resistance Gene, qacA
Source: Front Microbiol. 2018 Nov 19;9:2664. doi: 10.3389/fmicb.2018.02664 (PMC6252503; doi:10.3389/fmicb.2018.02664)
Supplement: Supplementary file 3 [file Table_3.docx]

Supplemental Table 3: Primer sequences for pC02 plasmid screen

| **Location on pC02 plasmid**  **(PCR designation)^a^** | **Primer** | **Primer Sequences (5'-3')** | **Reference** |
| --- | --- | --- | --- |
| 598-2098  (PCR1) | PP1-For | TGCCAATCATGGTATAGCTTCT | This study |
|  | PP1-Rev | GCTCGTCTAGTTCTTCGACT |  |
| 5833-7327  (PCR2) | PP2-For | ACTTGAACAGTGGCGATATGT | This study |
|  | PP2-Rev | GCTTGGAATTGTGGTGATCC |  |
| 14526-16001  (PCR3) | PP3-For | TGCCTCAACTAAGTGATGAAGAA | This study |
|  | PP3-Rev | TGCCCTCGTATTGAGGTATG |  |
| 19163-20570  (PCR4) | PP4-For | GAGGGCGTTATTATGCCAAA | This study |
|  | PP4-Rev | GCTTCATCAGCACCATGAAA |  |
| 23061-24338  (PCR5) | PP5-For | ACTCAACCCTTAATCCATCAACA | This study |
|  | PP5-Rev | TCAGCTTTGAAACAGGCATTC |  |
| 29792-31303  (PCR6) | PP6-For | GGGAGTAGCCTGTCTCATTT | This study |
|  | PP6-Rev | AGAGATTGTCCTGTTAATTCCCAT |  |
| 34577-36042  (PCR7) | PP7-For | CATTGAGTGCCTTTGCTGATAAA | This study |
|  | PP7-Rev | TCAAACCTAGTCATCCTTGCC |  |
| 50833-52183  (PCR8) | PP8-For | GCCCTTACAAAGCGATTACC | This study |
|  | PP8-Rev | ACACCTTCAGTGTACCATAACTT |  |
| 54906-56159  (PCR9) | PP9-For | AATAGATCAGTAACATCTGCTTGAG | This study |
|  | PP9-Rev | CGACTTCCTTGCCACTACT |  |
| 58751-60307  (PCR10) | PP10-For | GCAGACATCGTGCTGAAA | This study |
|  | PP10-Rev | CTCGTTCAGATTCATCTTGCTT |  |
| *qacA/B* | *qacAF* | GCTGCATTTATGACAATGTTTG | (81) |
|  | *qacAR* | AATCCCACCTACTAAAGCAG |  |

^a^ numbers indicate amplified nucleotide position as defined by the pC02 backbone.
