# Supplementary material for: Conjugative Transfer of a Novel Staphylococcal Plasmid Encoding the Biocide Resistance Gene, qacA
Source: Front Microbiol. 2018 Nov 19;9:2664. doi: 10.3389/fmicb.2018.02664 (PMC6252503; doi:10.3389/fmicb.2018.02664)
Supplement: Supplementary file 4 [file Data_Sheet_1.PDF]

Supplementary Material:
Origin and properties of particles found in sediment traps in Fram Strait

1 SUPPLEMENTARY FIGURES

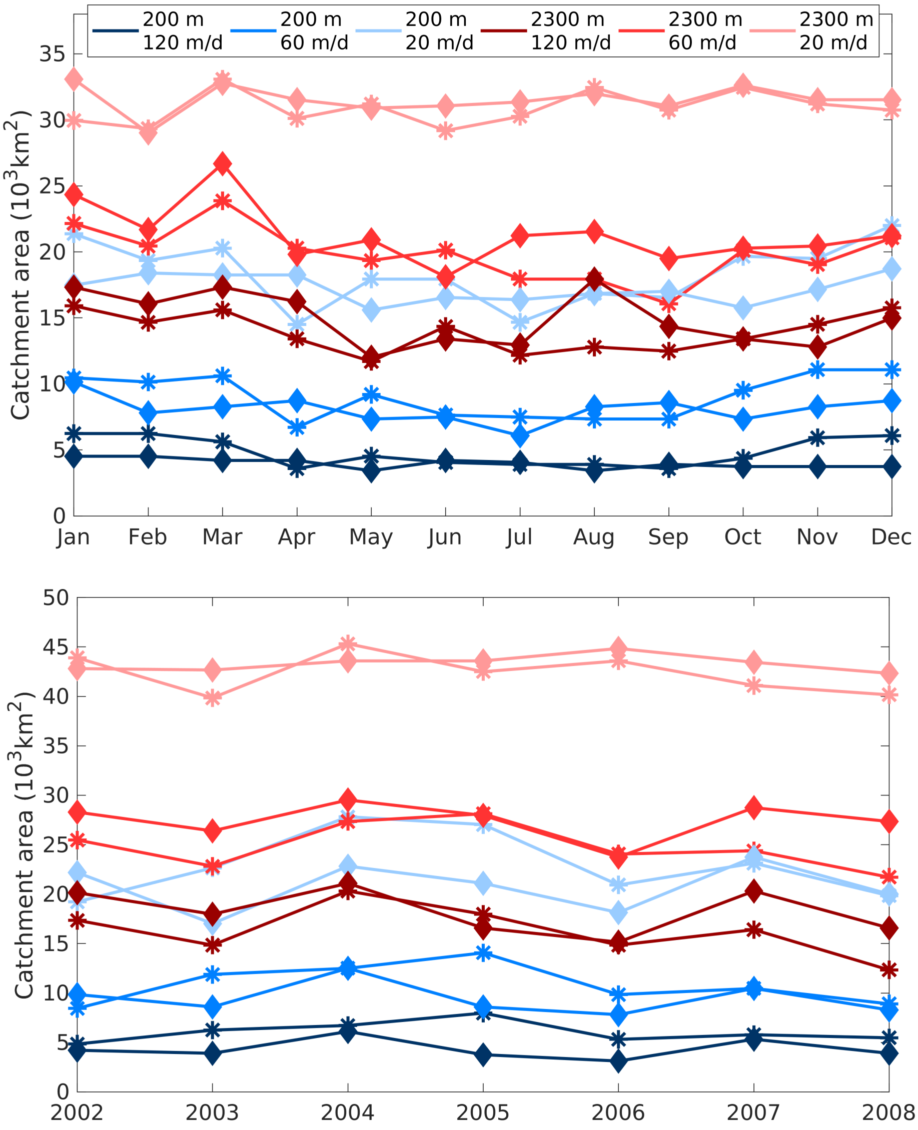

Figure S1. (Top) Seasonal cycle and (bottom) inter-annual variability of the spatial extent of the catchment area for sediment traps HG-N (indicated by star) and HG-IV (indicated by diamond). The spatial extent is calculated by adding up bins of 12.5 km x 12.5 km size in which at least one trajectory reaches the surface.

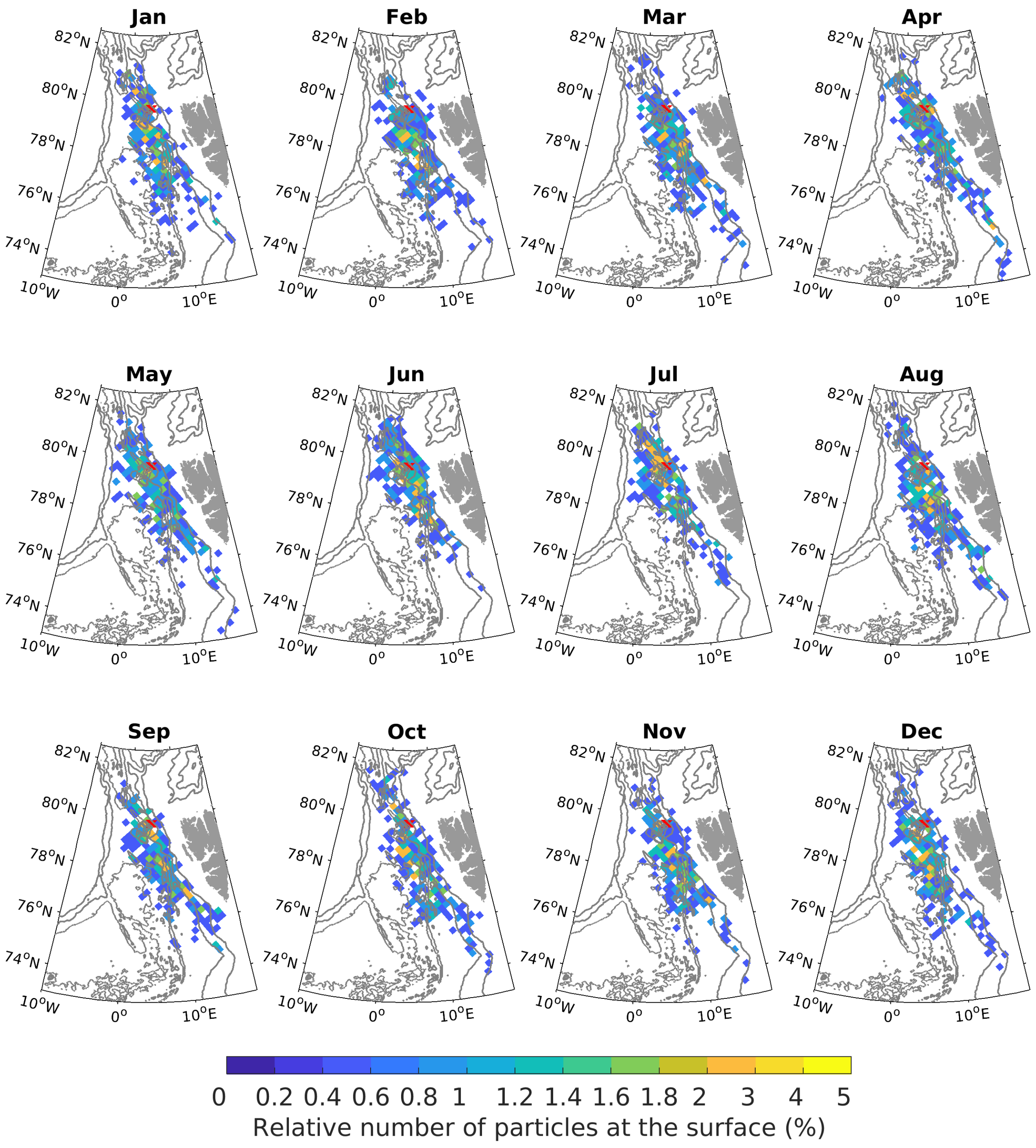

Figure S2. Relative number of particles (in %) reaching the surface in bins of 24 km x 24 km size for each month of the year averaged over the years 2002-2009, originating from mooring HG-N at 2300 m depth, using a sinking speed of 20 m/d. The red cross indicates the mooring location. Grey contours show the bathymetry at 1,000 m intervals.
