# Supplementary material for: Conjugative Transfer of a Novel Staphylococcal Plasmid Encoding the Biocide Resistance Gene, qacA
Source: Front Microbiol. 2018 Nov 19;9:2664. doi: 10.3389/fmicb.2018.02664 (PMC6252503; doi:10.3389/fmicb.2018.02664)
Supplement: Supplementary file 5 [file Data_Sheet_2.PDF]

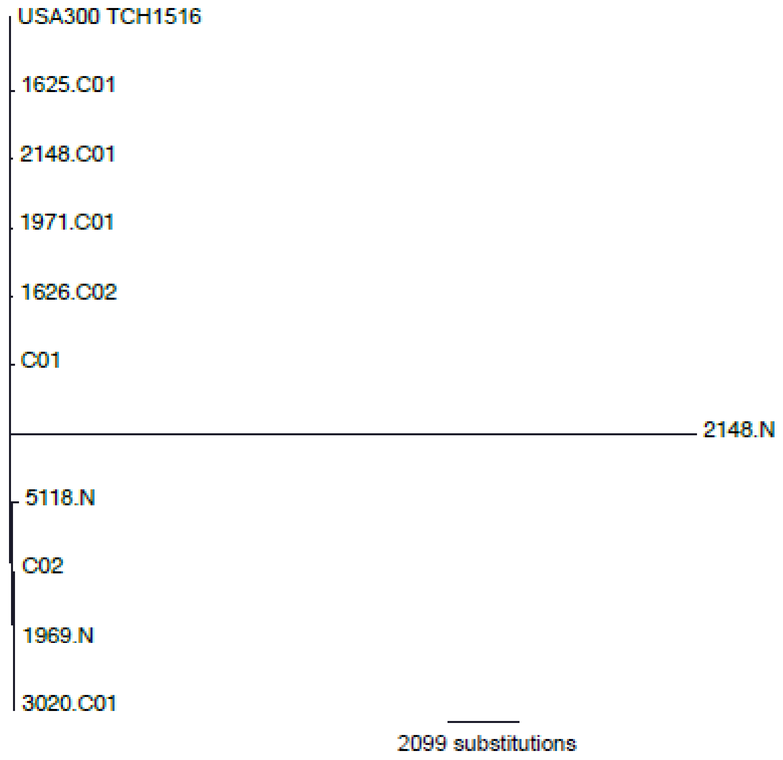


Supplemental Figure 2. Dendrogram of MRSA isolates that were subjected to whole genome sequencing and analysis in this study, prior to removal of the outlier *Staphylococcus aureus* 2148.N. A multiple sequence alignment (MSA) was created from nucleotide substitutions, small deletions called by GATK (McKenna et al., 2010), and putative large deletions where no reads mapped. A maximum likelihood tree was constructed with *S. aureus* USA300_TCH1516 as the root from the nucleic acid MSA using PhyML (Guindon et al., 2010).
